# Stressor-Induced “Inflammaging” of Vascular Smooth Muscle Cells via Nlrp3-Mediated Pro-inflammatory Auto-Loop

**DOI:** 10.3389/fcvm.2021.752305

**Published:** 2021-12-20

**Authors:** Jaqueline Herrmann, Mengdi Xia, Manasa Reddy Gummi, Anna Greco, Annika Schacke, Markus van der Giet, Markus Tölle, Mirjam Schuchardt

**Affiliations:** ^1^Department of Nephrology and Medical Intensive Care, Charité – Universitätsmedizin Berlin, Cooperate Member of Freie Universität and Humboldt Universität, Berlin, Germany; ^2^Department of Chemistry, Biochemistry and Pharmacy, Freie Universität Berlin, Berlin, Germany; ^3^Department of Nephrology, Second Clinical Medical Institution of North Sichuan Medical College (Nanchong Central Hospital), Nanchong, China

**Keywords:** calcification, senescence, inflammation, Nlrp3, smooth muscle cell

## Abstract

Calcification of the vessel wall as one structural pathology of aged vessels is associated with high cardiovascular mortality of elderly patients. Aging is linked to chronic sterile inflammation and high burden of reactive oxygen species (ROS), leading to activation of pattern recognition receptors (PRRs) such as Nlrp3 in vascular cells. The current study investigates the role of PRR activation in the calcification of vascular smooth muscle cells (VSMCs). Therefore, *in vitro* cell culture of primary rat VSMCs and *ex vivo* aortic stimulations were used to analyze osteogenic, senescence and inflammatory markers via real-time PCR, *in situ* RNA hybridization, Western Blot, photometric assays and histological staining. Induction of ROS and DNA-damage by doxorubicin induces a shift of VSMC phenotype toward the expression of osteogenic, senescence and inflammatory proteins. Induction of calcification is dependent on Nlrp3 activity. Il-1β as a downstream target of Nlrp3 induces the synthetic, pro-calcifying VSMC phenotype. Inhibition of PRR with subsequent reduction of chronic inflammation might be an interesting target for reduction of calcification of VSMCs, with subsequent reduction of cardiovascular mortality of patients suffering from vessel stiffness.

## Introduction

Cardiovascular disease (CVD) is the most critical age-related cause of death. Almost 70% of patients with new CVD are over 75 years old, so that the age might be the most prominent cardiovascular risk factor (1). One sign of structural pathology of the vessel wall associated with vascular aging is medial calcification, leading to increased vessel stiffness and pulse-wave velocity (2, 3). Medial calcification is common in patients with other comorbidities as e.g., chronic kidney disease (CKD) (2, 4, 5). Until now, no therapy exists to effectively reduce the increased cardiovascular risk associated with vessel calcification (3).

In particular involved in calcification of the medial layer of the vessel wall is the accumulation of senescent vascular smooth muscle cells (VSMCs) with a senescence-associated secretory phenotype (SASP) (6). Senescent cells express typical senescence markers [e.g., p53/p21, lysosomal senescence-associated β-galactosidase activity (SA-β-Gal), γH2A.X], and show an increased vulnerability to exogenous stressors (7–9). VSMCs physiologically have a contractile phenotype and express specific VSMC marker proteins (e.g., SM22α, Myh11, Cnn1, Acta2). Due to phenotype plasticity, VSMCs can adapt an osteoblast-like phenotype characterized by decreased expression of VSMC markers and increased expression of osteoblast markers e.g., core binding factor alpha-1 (Cbfa-1), tissue non-specific alkaline phosphatase (Alp), osteopontin (Opn), and bone morphogenetic protein-2 (Bmp-2) (9, 10). Recently, Shanahan's group pointed out that the DNA damage-induced calcification is dependent on activation of Cbfa1 in VSMCs (11). They found Cbfa1 to be involved in DNA damage response and therefore bridging osteogenic transition and apoptosis in the mineralization process (11).

During physiological aging, a chronic sterile inflammation, known as “inflammaging,” develops. This “inflammaging” is primarily based on mechanisms of the innate immune system that involve activation of pattern recognition receptors (PRRs) (12, 13). PRRs are a link between inflammation and cellular senescence. PRRs are mainly transmembrane toll-like receptors (Tlrs) and cytoplasmic Nod-like receptor (Nlr) inflammasomes (14). The Nlrp3 is the best characterized inflammasome and is expressed in VSMCs (15). The activation of Nlr inflammasomes induces the secretion of Il-1 cytokines e.g., Il-1β (16). Il-1β has strong pro-inflammatory effects by activating different Il-1 receptors (Il-1Rs). It induces the expression of other pro-inflammatory cytokines, especially Il-6, and can increase its own expression by a positive feedback mechanism (17). Cytokines and chemokines as major pro-inflammatory mediators can contribute to chronic inflammation and senescence (18).

Doxorubicin (DOX) is an anthracycline antitumor drug (19). DOX is known to induce several kinds of DNA damages by intercalation, generation of free radicals, DNA-binding, alkylation and cross-linking, DNA strands separation, influenced helicase activity and inhibition of topoisomerase II (19). Previous studies have not only investigated effects of DOX on stress-induced senescence (20) but also, induction of Alp activity upon DOX treatment; however, only after seven days of treatment (20). In addition, recent studies have shown the important role of DNA-damage response and the Cbfa1-dependent link to the calcification process of VSMCs (9, 11). Cobb et al. pointed out that the DNA damage-induced calcification is dependent on activation of Cbfa1 in VSMCs (11). They found Cbfa1 to be involved in DNA damage response and therefore bridging osteogenic transition and apoptosis in the mineralization process (11).

However, there are no detailed studies on the relationship of stress-induced senescence as e.g., caused by DOX, subsequent activation of Nlrp3-dependent pro-inflammatory signaling and calcification in VSMCs. We aim to show that stressor-induced senescence in VSMCs results in a pro-inflammatory response and induction of calcification. Accordingly, we used DOX as stressor. We are aware that DOX might not reflect the whole spectrum of senescence induction in VSMCs; however, DNA damage, upregulated under treatment with DOX, is a known and well-documented inducer of cellular senescence (9, 11).

Therefore, the current study investigates the effects of the stressor DOX on the initiation of processes of acute “inflammaging” and vascular calcification. The increase in calcification upon DOX treatment was dependent on the activation of the Nlrp3 inflammasome. Il-1β as Nlrp3 downstream effector amplifies its own expression. The initial stressor-induced acute “inflammaging” process then can be continued via a Nlrp3 inflammasome-mediated auto-inflammatory loop resulting in SASP and calcification of VSMCs.

## Materials and Methods

All cell culture components were obtained from Biochrom AG and Bio and Sell. DOX was obtained from Thermo Fisher. Recombinant rat Il-6 and Il-1β were purchased from PeproTech. VAS2870 was obtained from Sigma Aldrich and MCC950 from Invivogen. Tiron was purchased from Biozol and TAK242 from Biomol.

### Animals

All experiments with animals were done under minimal animal suffering and in accordance with the EU Directive 2010/63/EU. The experiments were approved by the Landesamt fuer Gesundheit und Soziales Berlin (T0211/02), Germany and the Charité - Universitätsmedizin Berlin, Germany.

Wistar rats were purchased from Janvier Labs. Nlrp3^−/−^ and Nlrp3^+/+^ (genetic background: C57BL/6) were bred at the Charité-Universitätsmedizin Berlin animal facility.

Euthanasia of animals was accomplished with intraperitoneal injection of sodium pentobarbital (rats: 400 mg/kg body weight, mice: 200 mg/kg body weight).

### VSMCs Cell Culture

Primary rat VSMCs from aortic tissue (aortic arch and thoracic aorta) of Wistar rats (mean age 4 months, male/female) were cultured by the outgrowth technique described previously (21). VSMCs at passages 3 to 7 were used for experiments. Cells were cultured in a humidified incubator at 37°C with 5% carbon dioxide. If not stated otherwise, VSMCs were cultured in Dulbecco Modified Eagle Medium (DMEM) containing 1 g/l glucose, supplemented with 10% fetal calf serum (FCS), penicillin (100 U/ml) and streptomycin (0.1 mg/ml) (culture medium). For gene expression, ROS detection, protein secretion and immunohistology experiments, cells were serum-starved for 24 h and stimulated using DMEM with 4.5 g/l glucose (w/o phenol red), supplemented with 1% glutamine, penicillin (100 U/ml) and streptomycin (0.1 mg/ml).

### Preparation of Aortic Rings for *ex vivo* Experiments

For *ex vivo* stimulation of aortic tissue, the adventitia was removed. Thoracic aortas of rats (mean age 4 months, male) or Nlrp3^−/−^ and Nlrp3^+/+^ mice (mean age 11 months, male/female) were dissected into aortic rings of equal size and incubated in a well-plate with the respective stimulation medium for 24 h or 14 days, respectively. Each stimulation contained several aortic rings from different aortic parts (aortic arch, different segments of descending aorta proximal to distal), which were equally distributed between stimulation and respective controls. The incubation procedure of the tissue took place in a humidified incubator at 37°C and 5% carbon dioxide.

### *In vitro* and *ex vivo* Calcification

Calcification was induced by exposing VSMCs or aortic rings (rat and mice) to DMEM containing 4.5 g/l glucose, supplemented with 15% FCS, 284 μmol/l ascorbic acid and 5 mmol/l inorganic phosphate, penicillin (100 U/ml) and streptomycin (0,1 mg/ml) [Calcification Medium (Calc M)]. As Control Medium (Ctrl M) served DMEM containing 4.5 g/l glucose, supplemented with antibiotics. Calcification was induced over 14 days of stimulation with Ctrl M, Calc M and in co-stimulation with DOX (10 or 100 nmol/l), Il-6 (100 ng/ml), Il-β (100 ng/ml) and MCC950 (50 μmol/l). Medium was replaced every two to three days.

### Gene Expression

VSMCs were serum-starved for 24 h prior to stimulation for 48 h. Cells were washed after stimulation with phosphate buffered saline (PBS) on ice and lysed with RLT™ cell lysis buffer (Qiagen). RNA was isolated according to the RNeasy™ Mini kit protocol (Qiagen). The RNA was reverse transcribed using the High-Capacity cDNA Reverse Transcription Kit™ (Applied Biosystems) according to the manufacturer's instructions. For the quantitative determination of mRNA expression, the iQ™ SYBR Green SuperMix and the CFX384 real-time PCR detection system (Biorad, CFX software version 3.1) were used. The oligonucleotides (Supplementary Table 1) were synthesized by TibMolBiol. Each sample was performed as technical duplicate for real-time PCR. β-actin and Gapdh were used as housekeeper genes for normalization. Analysis was performed with the ΔΔCT-method.

### Measurement of Alp Activity

After stimulation, VSMCs were lysed and scraped in 0.2% Triton X/PBS lysis buffer. Alp activity was assessed using a p-nitrophenyl phosphate-based Alp Assay Kit (Abcam) according to the manufacturer's recommendations. Protein content was determined with the bicinchoninic acid (BCA) protein assay kit (Pierce) and was used for normalization. Photometric measurements were conducted with a Multiskan Spectrum (Thermo Electron Corporation).

### Quantification of Calcium Content

For quantification of calcification, VSMCs or aortic rings were decalcified in 0.6 mol/l HCl overnight or for 24 h, respectively. After decalcification, cells were washed with PBS and lysed in 0.1 mol/l NaOH/0.1% SDS buffer. The protein content was quantified using BCA protein assay kit (Pierce). Aortic rings were dried and weighed. Calcium content was quantified using the colorimetrical o-cresolphthalein method (Colorimetric Calcium Assay, ScienCell) according to the manufacturer's recommendation. Photometric measurements were conducted with a Multiskan Spektrum (Thermo Electron Corporation). Protein content (*in vitro*) or aortic dry weight (*ex vivo*) was used for normalization, respectively.

### Histological Staining of Calcium Deposits

Upon stimulation of VSMCs for 14 days, the cells were fixed with 4% buffered formaldehyde, washed with PBS and distilled deionized water and treated with Alizarin Red solution (2%, pH 4.2) for 20 min, then washed again and imaged.

Upon stimulation of aortic rings for 14 days, the tissue was fixed overnight, transferred to 70% ethanol, and embedded in paraffin via automatic procedure. The aortas were serially cut into 4 μm sections, stained with Alizarin Red solution (0.5%, pH 4.2) and imaged. For all histological imaging, the Axiovert 200M microscope (Zeiss) with Zen2 software (Blue edition, Zeiss) was used.

### ROS Staining

VSMCs were seeded in 8-well slides (LabTec, Thermo Fisher, μ-slide, Ibidi), serum-starved for 24 h, and stimulated for 30 min as indicated. Afterwards, cells were washed and treated with 30 μmol/l dihydroethidium (DHE) (Molecular Probes) for 30 min. Cells were fixed with cold formalin (4%) for 5 min and subsequently washed with PBS. LabTec were mounted with ProLong™ Gold antifade mount (Thermo Fisher) and stored in the dark until imaging. For μ-slides, wells were covered with PBS and immediately imaged. A more detailed description of the staining procedure and data analysis can be found in the Supplementary Material. For all imaging of fluorescence stainings, an Axiovert 200M microscope (Zeiss) with Zen2 software (Blue edition, Zeiss) was used.

### Immunohistology and mRNA *in situ* Staining

VSMCs were stained for SA-β-Gal activity, histone γH2A.X, Bmp-2, Opn and p21 mRNA according to a previously published protocol (22) with some modifications. Briefly, cells were seeded in 8-well LabTec chamber slides (Thermo Fisher) or μ-slide (Ibidi), serum-starved for 24 h and stimulated for 48–72 h. Cells were stained for the desired target and imaged. A more detailed description of the staining procedure and data analysis can be found in the Supplementary Material. Quantification of fluorescence intensity was done with Zen2 (Blue edition, Zeiss) and Fiji/ImageJ.

### Western Blot

VSMCs were stimulated with DOX (500 and 1,000 nmol/l) and Il-1β (100 ng/ml) in cell culture medium for 48 h. For protein extraction, cells were washed with ice-cold PBS and lysed in cold RIPA buffer (Thermo Fisher Scientific). Protein content was assessed with BCA protein assay kit (Pierce). 15–10 μg protein per lane (p21: 15 μg, all others 10 μg) was applied on the respective gel. Protein samples were mixed and dissolved in 4× Laemmli buffer (Biorad) and heated to 95°C for 15 min. The proteins were separated on 12% TGX Gels (Biorad) and transferred onto a polyvinylidene difluoride membrane. The membranes were immunoblotted overnight at 4°C with primary antibodies: rabbit anti-p21 (1:2,500, ab109199, Abcam), rabbit anti-Alp (1:500, 7H11L3, Invitrogen), rabbit anti-Cbfa1 (1:1,1000, sc-10758, Santa Cruz), and mouse anti-β-actin (1:5,000 8H10D10, Cell Signaling Technology). After washing five times for five min each with TBST, the membranes were incubated with conjugated fluorescent secondary antibodies [anti-rabbit-StarBright Blue700 (1:2,500, #12004162, Biorad) and anti-mouse-StarBright Blue520 (1:2,500, #12005867)]. The bands were visualized using a ChemiDoc MP Imaging System (Biorad).

### Luminex™

VSMCs were serum-starved for 24 h prior to stimulation with Il-1β (100 ng/ml) and respective antagonists [VAS2870 (10 μM), MCC950 (50 μM), Tiron (10 mM), TAK242 (10 μM)] for 48 h. Rat aortic rings were stimulated with DOX (1000 nmol/l) and MCC950 (50 μM) for 24 h. After stimulation, supernatant was collected for cytokine quantification. Cytokine concentrations in the supernatant were determined using the Milliplex™ Cytokine Kit (Millipore) according to the manufacturer's instructions. Measurements were conducted using the Bio-Plex device and respective Bio-Plex software (version 6.1, Biorad). Cells were washed with cold PBS and lysed in NP40 buffer, followed by protein quantification using BCA protein assay kit (Pierce). Aortic rings were dried and weighed for normalization.

### Statistical Analysis

Data are provided as mean ± SEM of at least 3 independent experiments. Statistical analysis was performed using GraphPad Prism software (version 6.0). The one-way Anova with multiple comparisons or Wilcoxon matched paired test were applied to evaluate differences between treatment groups. A *p*-value < 0.05 was considered as statistically significant.

## Results

### Stressor-Induced Calcification With Osteogenic Transition

**Figure 1:**
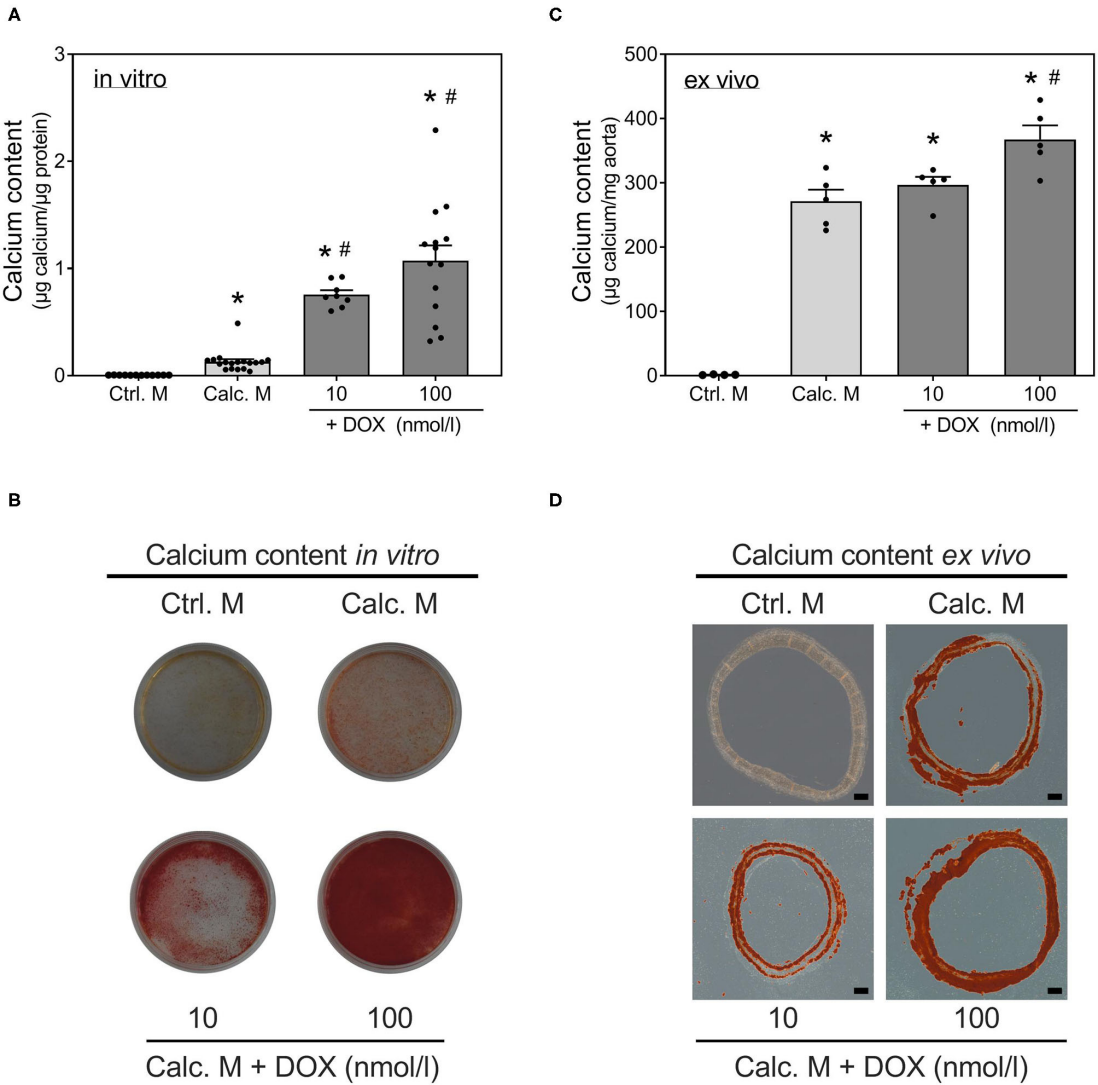
Induction of calcification with doxorubicin. VSMCs **(A,B)** or rat aortic rings **(C,D)** were stimulated with control medium or calcification medium supplemented with DOX as indicated for 14 d. Calcium content was quantified with o-cresolphthalein **(A,C)** and normalized to protein content (μg) **(A)** or aortic weight (mg) **(C)**. Calcium deposits were stained with Alizarin Red **(B,D)**. Data represent mean ± SEM, *n* ≥ 3, ^*^p < 0.05 vs. indicated control, #*p* < 0.05 vs. calcification medium. Pictures show one representative experiment of *n* ≥ 3 **(B,D)**, the scale bar indicates a 200 μm section **(D)**.

Stimulation with calcifying medium robustly induced calcification of VSMCs after 14 d (Figure 1A). Co-stimulation with DOX significantly and dose-dependently reinforced calcification of VSMCs *in vitro*: quantification (Figure 1A) shows a significant and dose-dependent induction of calcification under co-stimulation of calcification medium and DOX. This is also visualized by Alizarin Red staining (Figure 1B). To confirm these *in vitro* findings, an *ex vivo* setting using aortic rings was conducted. Calcium content of rat aortic rings was quantified upon 14 d of stimulation, and calcification was visualized via Alizarin Red staining (Figures 1C,D). DOX stimulation in calcifying medium dose-dependently increases calcium deposition *ex vivo*.

**Figure 2:**
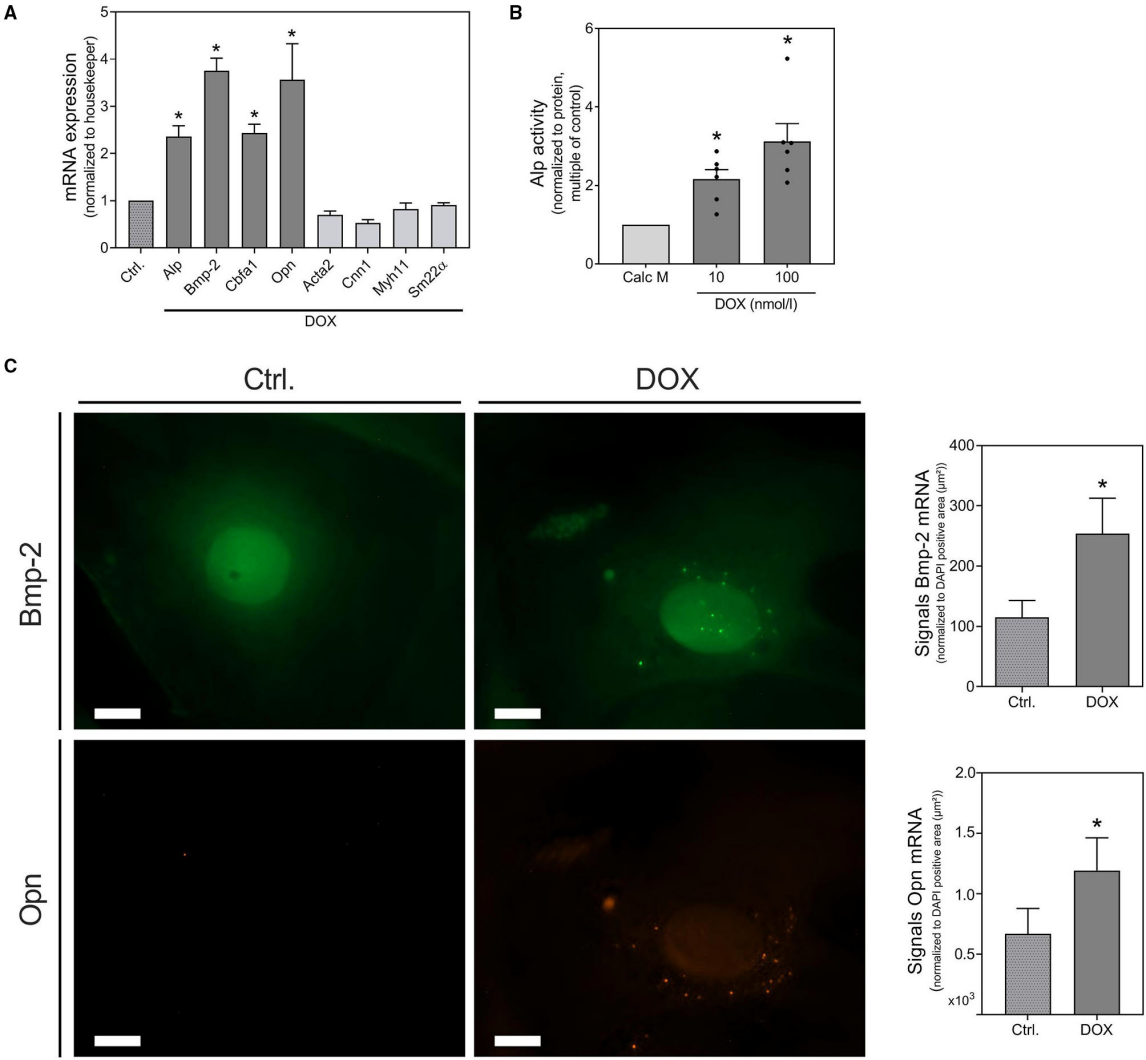
Induction of osteoblastic trans-differentiation by doxorubicin. VSMCs were stimulated in quiescence medium **(A,C)** or calcification medium **(B)** with DOX as indicated or 1,000 nmol/l for 48 h **(A,C)** or 14 d **(B)**. mRNA expression was measured with quantitative real-time PCR **(A)**. Measured thresholds of the respective targets were normalized to the corresponding threshold of housekeeper gene actin before normalization to control **(A)**. Alp activity was measured with a p-nitrophenyl phosphate-based Alp Assay and normalized to protein content (μg/μl) before normalization to control **(B)**. mRNA expression of Bmp-2 and Opn were visualized with RNA *in situ* hybridization staining and were quantified by counting signal dots per whole image, normalized to the DAPI positive cell core area per image (μm^2^) **(C)**. Data represent mean ± SEM, *n* ≥ 3, ^*^p < 0.05 vs. control. Pictures show a section of a representative experiment of *n* ≥ 3, the scale bar indicates a 10 μm section **(C)**. Full representative images are shown in Supplementary Table 3.

Further evidence for the pro-calcifying potential of DOX derives from mRNA detection of osteogenic markers as Bmp-2, Cbfa1 and Opn, all of them significantly increased upon DOX stimulation. VSMC marker as Acta2, Cnn1, Myh11, and SM22α slightly, but not significantly, decreased under stimulation with DOX (Figure 2A). The osteogenic transition is further confirmed by detection of Alp activity increase upon DOX treatment (Figure 2B). The induction of calcification markers after stimulation with DOX was confirmed by *in situ* hybridization analysis for Bmp-2 and Opn. Both markers were visualized via fluorescence staining and quantified by counting signal dots per whole image (Figure 2C). Although DOX influences cell behavior and growth, the cytotoxicity of DOX in our cell model did not reach statistical significance in the used setting (Supplementary Figure 1).

### Stressor-Induced Induction of “Inflammaging”

**Figure 3:**
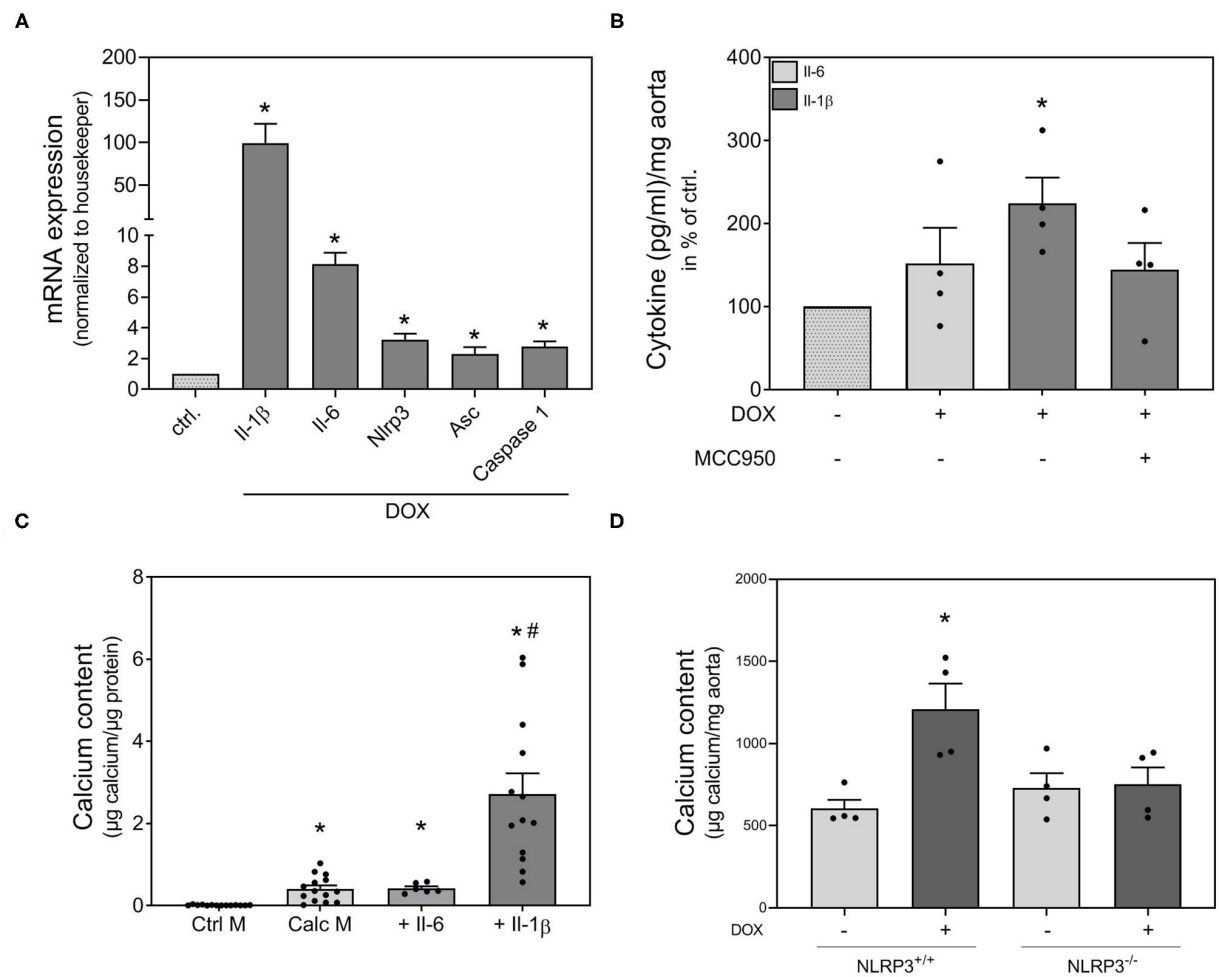
Involvement of Nlrp3 in doxorubicin induced calcification. VSMCs **(A,C)** were stimulated as indicated with 1,000 nmol/l DOX **(A)**, 100 ng/ml Il-1β **(C)** and 100 ng/ml Il-6 **(C)** for 48 h **(A)** or 14 d **(C)** in quiescence medium **(A)**, control medium (Ctrl. M) **(C)** or calcification medium (Calc M) **(C)**. Aortic rings from rats were stimulated in quiescence medium with 1,000 nmol/l DOX and MCC950 (50 μM) as indicated for 24 h **(B)**. Aortic rings from Nlrp3^−/−^ mice and respective controls were stimulated for 14 d in calcification medium with DOX (100 nmol/l) as indicated **(D)**. mRNA expression was measured with quantitative real time PCR **(A)**. Measured thresholds of the respective targets were normalized to the corresponding threshold of housekeeper gene actin before normalization to control **(A)**. Protein secretion was assessed with Luminex™ and normalized to aortic weight (mg) **(B)**. Calcium content was quantified with *o*-cresolphthalein **(C,D)** and normalized to protein content (μg) **(C)** or aortic weight **(D)**. Data represent mean ± SEM, *n* ≥ 3, ^*^p < 0.05 vs. control, #*p* < 0.05 vs. Calc M.

Il-1β and Il-6 are known essential components of the SASP (23). Therefore, we investigated the effect of DOX on the expression of both cytokines. DOX induced Il-1β and Il-6 mRNA expression in VSMCs (Figure 3A). It is already known from the literature that the Nlrp3 inflammasome is involved in the phenotype switching of VSMCs and in Il-β production (15). Therefore, we measured the mRNA expression of Nlrp3, its cofactor Asc and the associated enzyme caspase-1 that cleaves the preform of Il-1β (pre-Il-1β) into active Il-1β. The mRNA expression of all three proteins was significantly increased upon DOX stimulation (Figure 3A). Also, the secretion of Il-1β was significantly upregulated after DOX stimulation, while no significant secretion of Il-6 was found upon DOX treatment. The DOX-induced Il-1β secretion was reduced by co-stimulation with Nlrp3 inhibitor MCC950 (Figure 3B). As Il-1β and Il-6 are both components of the SASP, we tested the pro-calcifying potential of both substances and found Il-1β to significantly induce calcification, whereas Il-6 did not exhibit calcifying potential in our model (Figure 3C). To validate the role of the Nlrp3 inflammasome for calcification, we used an *ex vivo* setting with aortic rings from Nlrp3^−/−^ and respective control mice (Nlrp3^+/+^). While DOX significantly induced aortic calcification in Nlrp3^+/+^, the effect is lost in aortas from Nlrp3^−/−^ mice (Figure 3D).

**Figure 4:**
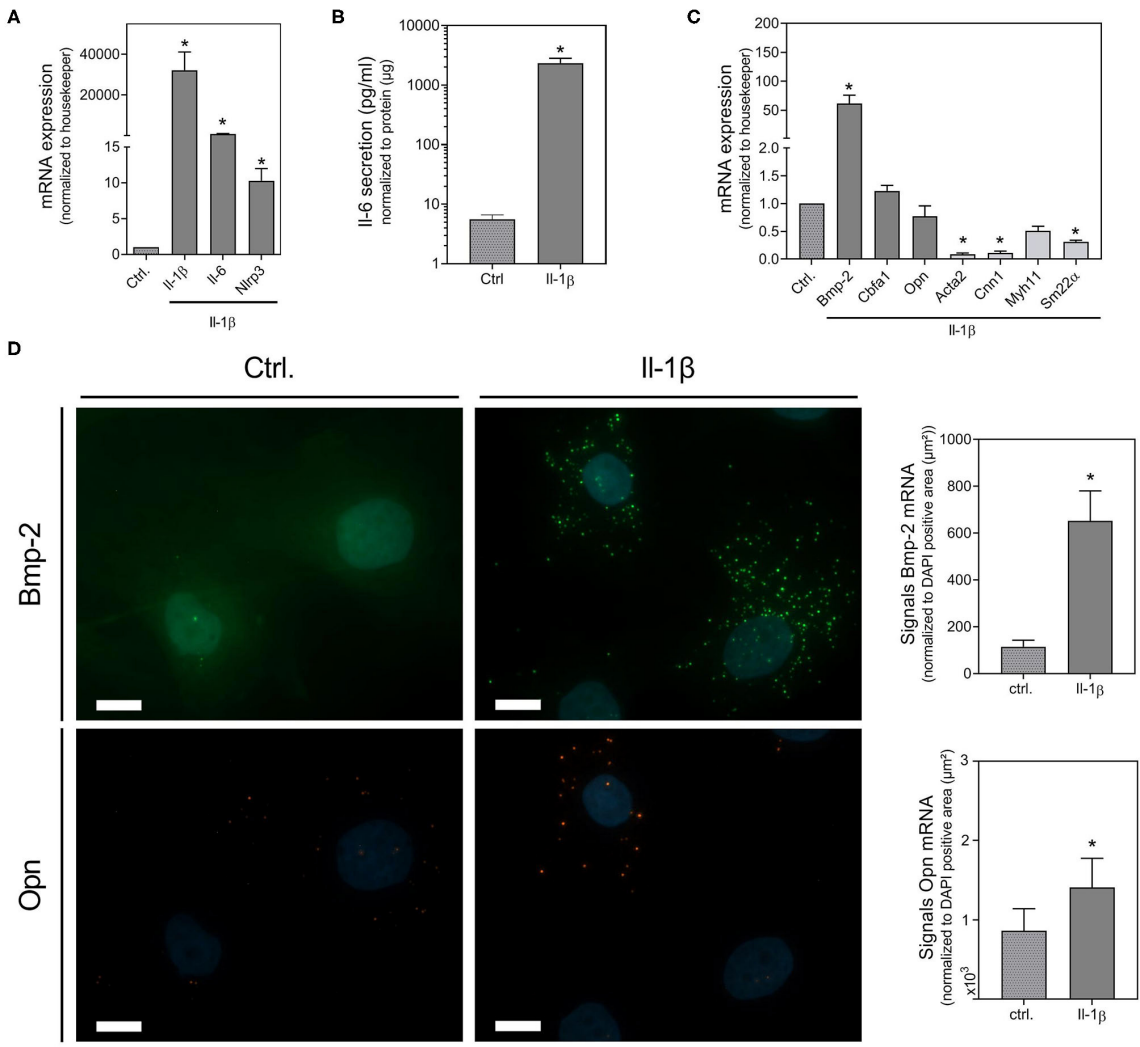
Il-1β induced changes in osteoblastic and pro-inflammatory gene expression and protein secretion. VSMCs were stimulated for 48 h with 100 ng/ml Il-1β. mRNA expression was measured with quantitative real-time PCR **(A,C)**. Measured thresholds of the respective targets were normalized to the corresponding average threshold of housekeeper genes actin and Gapdh before normalization to control **(A,C)**. Secretion of Il-6 was measured with Luminex™ and normalized to protein content (μg) **(B)**. Expression of Bmp-2, Opn were visualized with RNA *in situ* hybridization staining and were quantified by counting signal dots per whole image, normalized to the DAPI positive cell core area per image (μm^2^) **(D)**. Data represent mean ± SEM, *n* ≥ 3, ^*^p < 0.05 vs. control, pictures show a section of a representative experiment of *n* ≥ 3, the scale bar indicates a 10 μm section **(D)**. Full representative images are shown in Supplementary Table 4.

To further verify the involvement of Il-1β as effector molecule of the Nlrp3 inflammasome and of Il-6, we investigated their effect on mRNA expression of inflammation, senescence and calcification markers using real-time PCR and *in situ* hybridization. Il-1β significantly induces the expression of Il-6 and, in the context of a very potent pro-inflammatory auto-loop, Il-1β itself. In addition, Il-1β induces the expression of Nlrp3 (Figure 4A). Upon Il-1β treatment, VSMCs release Il-6 (Figure 4B). While the Il-1β-induced expression of the osteogenic marker Bmp-2 increased, the VSMCs marker SM22α, Acta2, and Cnn1 decreased upon Il-1β (Figure 4C). The increased mRNA expression of Bmp-2 upon Il-1β stimulation was confirmed by *in situ* hybridization technique. While for Opn mRNA expression via real-time PCR no significant effect could be detected, the *in situ* hybridization found a slight induction (Figure 4D).

**Figure 5:**
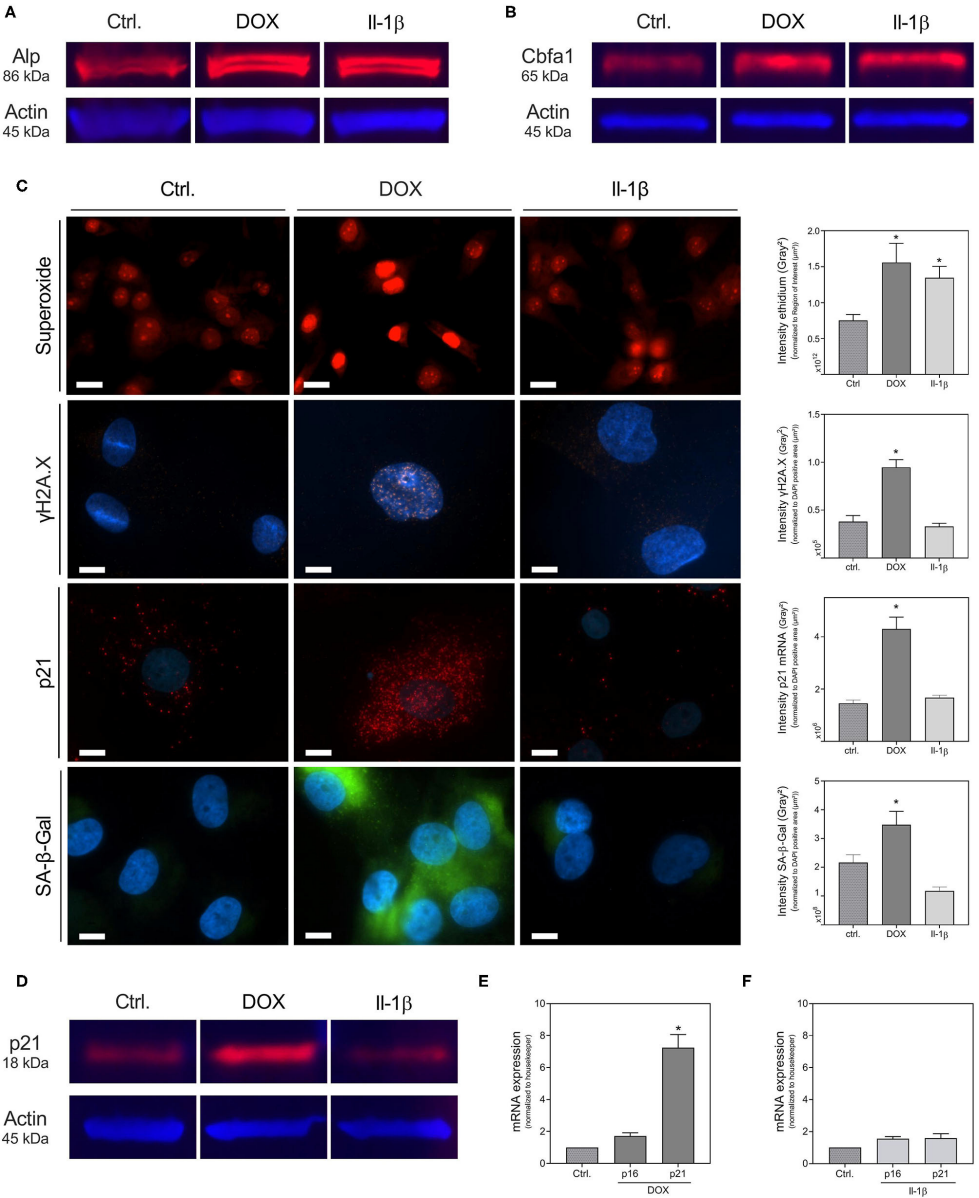
Formation of calcification markers, reactive oxygen species and senescence upon DOX and Il-1β treatment. VSMCs were stimulated for 30 min (superoxide), 48 h [**(A,B)**, γH2A.X, p21 *in situ* **(D–F)**], or 72 h (SA-β-Gal) with 100 ng/ml Il-1β or 1000 nmol/l DOX in quiescence medium **(C,E,F)** or culture medium **(A,B,D)**. Formation of superoxide was assessed with DHE, expression of γH2A.X was analyzed immunohistochemically, mRNA expression of p21 was assessed with RNA *in situ* hybridization and formation of SA-β-Gal was assessed with Spider staining **(C)**. For quantification of the expression of γH2A.X, mRNA expression of p21 and formation of SA-β-Gal, intensity of the respective fluorescence signal was measured after application of a threshold procedure as described in the supplement and the signal intensity then normalized to the DAPI positive cell core area per image (μm^2^). For quantification of DHE, regions of interest were detected with a threshold procedure as described in the supplement and measured intensity was normalized to the area of interest detected by the threshold procedure. Alp protein, Cbfa1 protein and p21 protein were assessed with Western Blot **(A,B,D)**. mRNA expression was measured with quantitative real-time PCR **(E,F)**. Measured thresholds of the respective targets were normalized to actin **(E)** or the corresponding average threshold of housekeeper genes actin and Gapdh **(F)** before normalization to control. Data represent mean ± SEM, *n* ≥ 3, *p < 0.05 vs. Control. The Western Blots show a representative experiment of *n* = 3. Full blots are shown in Supplementary Figures 3–5. Pictures show a section of a representative experiment of *n* ≥ 3, the scale bar indicates a 10 μm section (γH2A.X, p21, SA-β-Gal) or a 20 μm section (superoxide). Full representative images are shown in Supplementary Tables 3–5.

As already shown in Figures 1–4, both DOX and Il-1β induce several markers of calcification. In addition, the protein expression of Cbfa1 and Alp, detected via Western Blot, is increased upon DOX and Il-1β, respectively (Figures 5A,B). However, the effects of Il-1β and DOX on senescence markers differ in our experimental model. As shown in Figure 5C, ROS (superoxide production) is significantly increased by DOX and Il-1β. ROS are one inducer of DNA damage, which can result in double-strand DNA breaks. A marker of double-strand DNA breaks, the formation of γH2A.X, was significantly increased upon DOX treatment, while it is not affected by Il-1β stimulation (Figure 5C). In line, while DOX stimulation results in upregulated expression of the senescence markers p21 and SA-β-Gal, both were not induced by Il-1β (Figure 5C). This could be confirmed by p21 protein detection via Western Blot (Figure 5D). mRNA detection via real-time PCR confirmed the findings of increased p21 mRNA expression upon DOX (Figure 5E) and no effect on p21 expression upon Il-1β treatment (Figure 5F). Neither DOX nor Il-1β induced p16 mRNA expression in our cell model (Figures 5E,F).

### Involved Signaling Pathway

**Figure 6:**
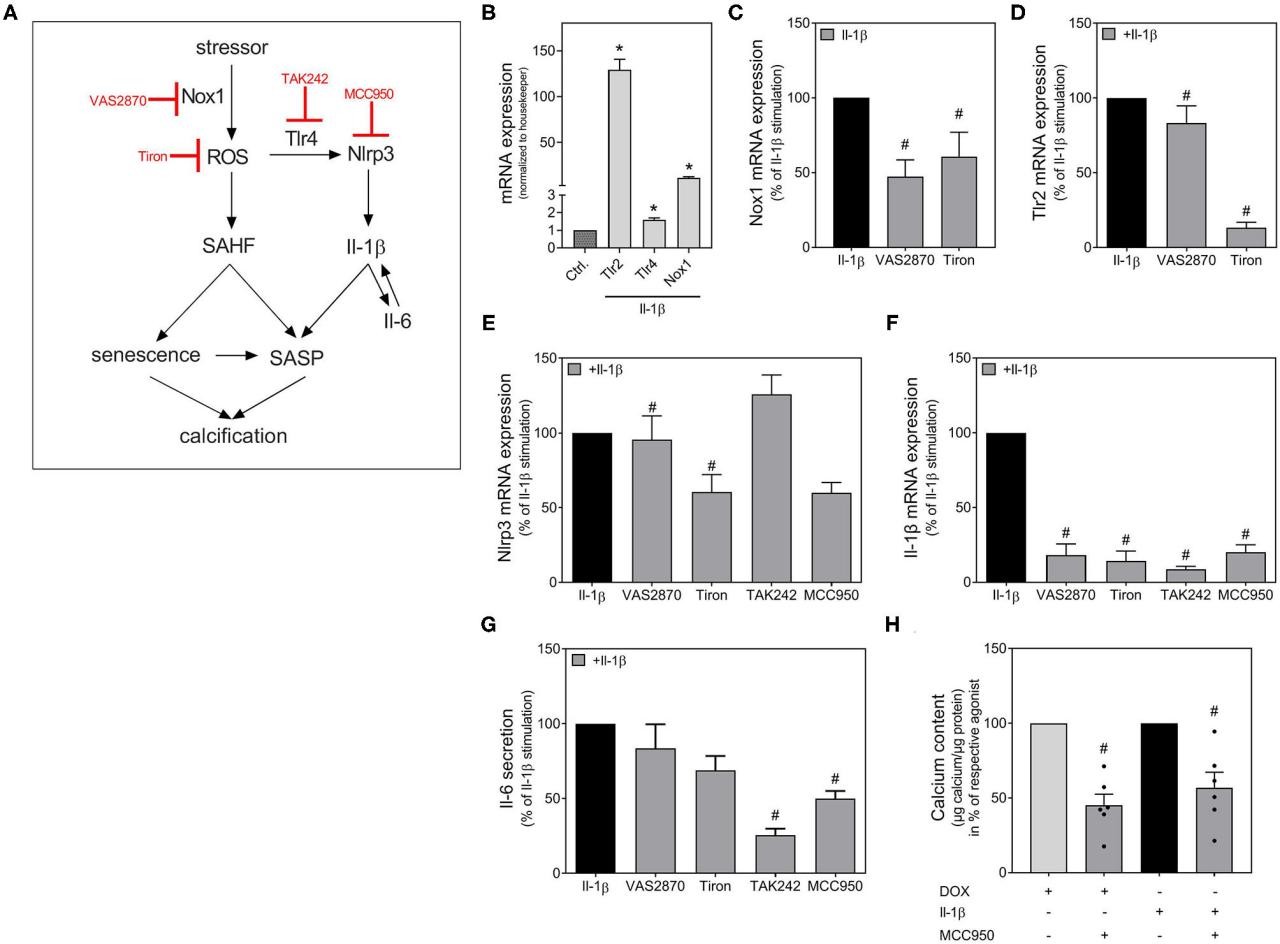
Possible signaling pathways in Nlrp3 dependent calcification. Scheme with inhibitors used **(A)**. VSMC were stimulated for 48 h **(B–G)** or 14 d **(H)** with Il-1β (100 ng/ml), DOX (100 nmol/l), VAS2870 (10 μM), MCC950 (50 μM), TAK242 (10 μM) or Tiron (10 mM) as indicated. mRNA expression was measured with quantitative real-time PCR **(B–F)**. Measured thresholds of the respective targets were normalized to the corresponding average threshold of housekeeper genes actin and Gapdh before normalization to control **(B–F)**. Secretion of Il-6 was measured with Luminex™ and normalized to protein content (μg) **(G)**. Calcium content was quantified with *o*-cresolphthalein and normalized to protein content (μg) **(H)**. Data represent mean ± SEM, *n* ≥ 3, ^*^p < 0.05 vs. Control, ^#^*p* < 0.05 vs. Respective agonist (100 ng/ml Il-1β or 100 nmol/l DOX).

As Il-1β strongly stimulates its own expression, we tested possible mediators of the signaling pathway involved in Il-1β-induced SASP activation. A schema of the Nlrp3 inflammasome activation and the therefore used antagonists is provided in Figure 6A. MCC950 was used as Nlrp3 inhibitor. It is already known from the literature that Tlrs work as one initial stimulus for Nlrp3 assembly and that the activation of Tlr2 and Tlr4 are involved in Il-1β release (24, 25). A previous study by our own working group has shown that the Tlr4 is constitutively active in our VSMCs cell model, whereas the Tlr2 is subsequently activated (26). Therefore, we tested the Tlr4 inhibitor TAK242. As increased ROS production serves as second stimulus for Nlrp3 activation (25), we also tested tiron as ROS scavenger. In VSMCs, the NADPH oxidase is one of the leading ROS producers (27). Therefore, the Nox1 inhibitor VAS2870 was also used. Both, Tlr2 as well as Tlr4 expression (first stimulus) and Nox1 expression (second stimulus), were induced in VSMCs upon Il-1β stimulation (Figure 6B). Both signals for Nlrp3 assembly could be significantly diminished by co-treatment with VAS2870 and tiron (Figures 6C,D). Nlrp3 mRNA expression itself was also diminished by tiron and MCC950 co-treatment, while VAS2870 and TAK242 have no significant effect (Figure 6E). Downstream, the Il-1β expression is significantly blocked by VAS2870, tiron, TAK242, and MCC950 (Figure 6F). The secretion of Il-6 could be significantly reduced by inhibition of ROS via tiron and Nlpr3 inhibition via MCC950 (Figure 6G). To verify the role of the Nlrp3-dependent pathway, we also tested Nlrp3 inhibition with MCC950 for calcification as endpoint. As shown in Figure 6H, both the DOX-and Il-β-induced calcifications are significantly inhibited by MCC950 co-treatment.

## Discussion

In the current study, we investigated the effect of ROS induction, DNA damage, and inflammation on the process of cellular senescence and calcification in *in vitro* and *ex vivo* models using rat VSMCs and thoracic aortas from rats and mice. After induction of ROS and DNA-damage by the primary stressor, the Nlrp3 inflammasome is activated with a subsequent auto-inflammatory loop driven by its effector molecule Il-1β.

As primary stressor molecule for VSMCs the anthracycline DOX was used. It has already been shown in literature that DOX causes DNA damage via induction of double strand breaks and ROS and promotes cellular senescence (20). Bielak-Zmijewska et al. investigated several markers of senescence in VSMCs upon DOX treatment (20). Although DOX might not reflect the whole spectrum of senescence induction in VSMCs, DNA damage is a known and well-documented inducer of cellular senescence (9, 11). As shown recently by Shanahan's group, the formation of nuclei positive for γH2A.X and ATM in VSMCs is induced by calcium phosphate and correlates with the induction seen by other DNA damagers as DOX and hydrogen peroxide treatments (9). γH2A.X is a marker of double-strand DNA breaks, which are specific domains of facultative heterochromatin that contribute to silencing of genes promoting proliferation in senescent cells and are one known marker of senescence-associated heterochromatin foci (SAHF) (28). The phosphorylation of γH2A.X by ATM is a key response mechanism to DNA damage, which can result in the activation of senescence pathways, including p21 (29). In line, in our experimental model treatment with DOX also induces DNA damage as γH2A.X and increases senescence markers p21 and SA-β-Gal. SA-β-Gal catalyzes the hydrolysis of β-galactosidase to monosaccharides in senescent cells. Cellular senescence is a dynamically regulated process, with activation of the p53/p21 and p16 pathways (30). In our experimental setting the p53/p21 pathway was upregulated by DOX, whereas p16 was not significantly affected (mRNA expression). While the senescence growth arrest via the p16 pathway seems not reversible, the p53-induced growth arrest is reversible upon p53 inactivation (31).

As in the previous study by Bielak-Zmijewska et al. only limited markers and only seven days of stimulation were investigated to study induction of calcification by DOX (20), we aimed to investigate DOX as primary stressor of DNA damage and senescence in order to analyze pro-inflammatory response and induction of calcification in VSMCs. In our experimental setting using primary VSMCs from rat thoracic aortas, DOX induces mineralization of VSMCs detected via calcium quantification and Alizarin Red staining after 14 days. The calcification induction was verified in an *ex vivo* setting using thoracic aortic rings. Analysis of the mRNA expression via real-time PCR and partly also *in situ* hybridization, enzyme activity and Western Blot confirmed the findings of osteogenic transformation of VSMCs; osteogenic markers as Cbfa1, Alp, Bmp-2, and Opn increase upon DOX treatment.

Senescent cells also have a metabolic active and pro-inflammatory phenotype termed as SASP. Cytokines of the Il-1 family and Il-6 are known to be part of this SASP (32). Furthermore, the induction of senescence by cytokines of the Il-1 family, e.g., Il-1β was investigated in cell culture and animal models (33). A microarray study has shown that senescent VSMCs reveal differential regulation of Matrix Gla Protein, Bmp-2, Osteoprotegerin, and Il-1β (34). In our own previous work, we demonstrated that plasma concentrations of Il-1β and Il-6 were significantly increased in rats treated with azathioprine, another known cellular stressor and ROS inducer, over a 24-weeks period of treatment (35). Increased expression of Il-1β and Il-6 was also detected in the aortas of treated rats, which were associated with increased expression of the osteogenic proteins Bmp-2, Alp and Cbfa1 (35). Therefore, we examined the effect of DOX on the expression of Il-1β and Il-6. The mRNA expression of Il-1β and Il-6 were significantly increased upon DOX stimulation, with higher induction for Il-1β. The Nlrp3 inflammasome, the most widely characterized inflammasome and known to be expressed in VSMCs (15), serves as most significant source of Il-1β production (16). Beside Nlr inflammasomes, transmembrane Tlrs are the main types of PRRs (14). The activation of PRRs mainly induces sterile inflammation associated with extensive transcriptional pro-inflammatory cellular reprogramming (36). This is temporarily useful in acute situations, while chronic activation is detrimental (37). Due to their potent pro-inflammatory potency, the Nlrp3 activation and assembly is a strictly controlled process with an initial stimulus e.g., activation of Tlr, and a second stimulus e.g., increased production of ROS (25). Subsequent activation of caspase-1 leads to cleavage and activation of pro-Il-1β into its active form Il-1β (25). PRRs are a link between the innate immune system, inflammation, and cellular senescence. VSMCs express small inflammasomes with low and slow onset, but long-lasting activity leading to a continuous release of Il-1 cytokines (15, 38). This mainly induces cellular hyperactivity and chronic sterile inflammation, which can maintain itself independently of the initial trigger (39). The cells retain their full viability and include Il-1 cytokines in the repertoire of their pro-inflammatory secretome (39). It was already shown that inflammasomes are involved in the calcification process and age-related diseases (40–42). In calcified VSMCs, Nlrp3, its cofactor Asc, and the cleavage enzyme caspase-1 are upregulated with subsequent Il-1β production, while Nlrp3 inhibition prevents *in vitro* calcification (38). In our experimental model, the expression of Nlrp3, Asc as well as caspase-1 is significantly upregulated upon DOX stimulation in VSMCs. Simultaneously, the expression of Tlr2 and Nox1 also increases. The NADPH oxidase, with Nox1 as one subunit, is one of the major sources for superoxide anion release in VSMCs (43). In VSMCs, the Tlr4 is constitutively active, while the Tlr2 is upregulated upon an inflammatory response (26).

As already known from a previous study by Wen et al., Il-1β can promote osteogenic differentiation and induction of calcification of VSMCs (38). In line, in our experimental setting, both Il-1β and DOX induce VSMC calcification. The DOX-induced mineralization could be significantly reduced by MCC950. This could be confirmed in the *ex vivo* setting with Nlrp3^−/−^ mice, where the calcification induction by DOX is also inhibited compared to control mice. This finding is comparable to our previous results regarding the stressor azathioprine, whose calcifying effect could also no longer be seen in aortic rings from Nlrp3^−/−^ mice (35). Therefore, Il-1β appears to be a very important effector cytokine, especially for the maintenance of a SASP after primary initiation of the SASP by the induction of “inflammaging” due to a cellular stressor. Thus, Il-1 cytokines might become an essential part of the respective pathogenesis by forming an auto-inflammatory loop, independent of the initial trigger.

Il-1β increases its own expression as well as the expression and secretion of Il-6. Moreover, Il-1β also enhances the expression of relevant components of the signaling pathway, particularly Tlr2, Nox1 and Nlrp3, also shown to be involved in DOX-mediated “inflammaging.” Yet, in contrast to the DOX-induced effects in VSMCs, Il-1β does not affect the expression of senescence markers, such as p21 or SA-β-Gal, nor induces DNA breaks detected via γH2A.X in our model using primary rat VSMCs. In contrast, a recent study found induction of senescence in a co-treatment of Il-1β and phosphate in human umbilical cord VSMCs (42). Different effects on calcification were also seen for Il-6 when the origin of VSMCs differs: while pro-calcifying effects of Il-6 were found in human umbilical artery VSMCs in non-osteogenic medium (44), others found these effects only in co-stimulation with the soluble Il-6 receptor in osteogenic medium in human VSMCs (45). At least one explanation might be the different origin of VSMCs that differ in protein expression, as demonstrated by proteomic analysis (46). Like Il-1β, processes of cellular senescence are not affected by Il-6 in our model (Supplementary Material), whereas an Il-6 production itself is a component of the SASP and therefore a sign of VSMCs senescence (32).

This study has some limitations that might hamper the direct translational aspects of the results: It was performed only in *in vitro* and *ex vivo* settings using cells and thoracic aortas from rats and mice. Further *in vivo* studies verifying the major role of Nlrp3 and Il-1β as a potential therapeutic target for the treatment of vessel “inflammaging” are necessary. In addition, the transferability in the current study is not shown for human aortic or other vessel beds.

In conclusion, the inhibition of PRRs could represent an essential approach for the therapy of systemic vascular diseases. The CANTOS study demonstrated for the first time in humans the importance of chronic vascular inflammation for CVD and the association of cardiovascular mortality with signs of systemic inflammation (47). The current study results provide further indications of a potential benefit of an interruption of the Nlrp3-associated auto-inflammatory loop.

## Data Availability Statement

The original contributions presented in the study are included in the article/Supplementary Material, further inquiries can be directed to the corresponding author/s.

## Ethics Statement

The animal study was reviewed and approved by Landesamt für Gesundheit und Soziales Berlin, Germany.

## Author Contributions

MS, MT, and MvdG: conceptualization, supervision, and project administration. JH, MS, and MT: methodology, writing—original draft preparation, and visualization. JH, MS, MX, MG, AG, MS, and AS: investigation. JH, MS, MT, and MvdG: data curation. JH, MT, MG, MX, AG, AS, and MvdG: writing—review and editing. MT, JH, MX, and MS: funding acquisition. All authors contributed to the article and approved the submitted version.

## Funding

This research was funded by a grant from the Bundesministerium für Bildung und Forschung (MT, MS), the Sonnenfeld Stiftung (MS, MT, JH) and the Berlin Institute of Health (MS). MX received a research scholarship of the Nanchong school science and technology strategic cooperation project (grant number: 20SXQT0117).

## Conflict of Interest

The authors declare that the research was conducted in the absence of any commercial or financial relationships that could be construed as a potential conflict of interest.

## Publisher's Note

All claims expressed in this article are solely those of the authors and do not necessarily represent those of their affiliated organizations, or those of the publisher, the editors and the reviewers. Any product that may be evaluated in this article, or claim that may be made by its manufacturer, is not guaranteed or endorsed by the publisher.

## Abbreviations

Acta2: Actin alpha 2
Alp: Alkaline Phosphatase
Asc: Apoptosis-Associated Speck-like Protein Containing a Caspase-Recruitment Domain
ATM: Ataxia–Telangiectasia Mutated
BCA: Bicinchonic Acid
Bmp-2: Bone Morphogenetic Protein-2
Calc M: Calcification Medium
Cbfa-1: Core Binding Factor alpha 1
Cnn1: Calponin
Ctrl M: Control Medium
CVD: Cardiovascular Disease
DAMP: Damage Associated Molecular Pattern
DHE: Dihydroethidium
DMEM: Dulbecco's Modified Eagle Medium
DOX: Doxorubicin
FCS: Fetal Calf Serum
Il-1β: Interleukin 1 β
Il-6: Interleukin 6
Myh11: Myosin Heavy Chain 11
Nlr: Nod-like Receptor
Nlrp3: Nod-like receptor family pyrin domain containing 3
Opn: Osteopontin
PBS: Phosphate Buffered Saline
PRRs: Pattern Recognition Receptors
ROS: Reactive Oxygen Species
Tlr: Toll-like Receptor
SA-β-Gal: Senescence Associated β-Galactosidase
SASP: Senescence Associated Secretory Phentoype
Sm22α: Smooth Muscle Protein 22-alpha
VSMCs: Vascular Smooth Muscle Cells.

## References

[1] Virani SS Alonso A Aparicio HJ Benjamin EJ Bittencourt MS Callaway CW. Heart disease and stroke statistics-2021 update: a report from the American heart association. Circulation. (2021) 143:e254–743. 10.1161/CIR0000000000000950 33501848 PMC13036842

[2] Tolle M Reshetnik A Schuchardt M Hohne M Van Der Giet M. Arteriosclerosis and vascular calcification: causes, clinical assessment and therapy. Eur J Clin Invest. (2015) 45:976–85. 10.1111/eci.12493 26153098

[3] Lanzer P Hannan FM Lanzer JD Janzen J Raggi P Furniss D. Medial arterial calcification: JACC state-of-the-art review. J Am Coll Cardiol. (2021) 78:1145–65. 10.1016/j.jacc.2021.06.049 34503684 PMC8439554

[4] Iribarren C Sidney S Sternfeld B Browner WS. Calcification of the aortic arch: risk factors and association with coronary heart disease, stroke, and peripheral vascular disease. JAMA. (2000) 283:2810–5. 10.1001/jama.283.21.2810 10838649

[5] Raggi P. Cardiovascular disease: coronary artery calcification predicts risk of CVD in patients with CKD. Nat Rev Nephrol. (2017) 13:324–6. 10.1038/nrneph.2017.61 28480902

[6] Childs BG Durik M Baker DJ Van Deursen JM. Cellular senescence in aging and age-related disease: from mechanisms to therapy. Nat Med. (2015) 21:1424–35. 10.1038/nm.4000 26646499 PMC4748967

[7] Kooman JP Kotanko P Schols AM Shiels PG Stenvinkel P. Chronic kidney disease and premature ageing. Nat Rev Nephrol. (2014) 10:732–42. 10.1038/nrneph.2014.185 25287433

[8] Munoz-Espin D Serrano M. Cellular senescence: from physiology to pathology. Nat Rev Mol Cell Biol. (2014) 15:482–96. 10.1038/nrm3823 24954210

[9] Sanchis P Ho CY Liu Y Beltran LE Ahmad S Jacob AP. Arterial “inflammaging” drives vascular calcification in children on dialysis. Kidney Int. (2019) 95:958–72. 10.1016/j.kint.2018.12.014 30827513 PMC6684370

[10] Herrmann J Babic M Tolle M Van Der Giet M Schuchardt M. Research models for studying vascular calcification. Int J Mol Sci. (2020) 21:2204. 10.3390/ijms21062204 32210002 PMC7139511

[11] Cobb AM Yusoff S Hayward R Ahmad S Sun M Verhulst A. Runx2 (Runt-Related Transcription Factor 2) links the DNA damage response to osteogenic reprogramming and apoptosis of vascular smooth muscle cells. Arterioscler Thromb Vasc Biol. (2021) 41:1339–57. 10.1161/ATVBAHA.120.315206 33356386

[12] Childs BG Li H Van Deursen J.M. Senescent cells: a therapeutic target for cardiovascular disease. J Clin Invest. (2018) 128:1217–28. 10.1172/JCI95146 29608141 PMC5873883

[13] Krainer J Siebenhandl S Weinhausel A. Systemic autoinflammatory diseases. J Autoimmun. (2020) 109:102421. 10.1016/j.jaut.2020.102421 32019685 PMC7610735

[14] Cao X. Self-regulation and cross-regulation of pattern-recognition receptor signalling in health and disease. Nat Rev Immunol. (2016) 16:35–50. 10.1038/nri.2015.8 26711677

[15] Sun HJ Ren XS Xiong XQ Chen YZ Zhao MX Wang JJ. NLRP3 inflammasome activation contributes to VSMC phenotypic transformation and proliferation in hypertension. Cell Death Dis. (2017) 8:e3074. 10.1038/cddis.2017.470 28981106 PMC5680591

[16] Tangi TN Elmabsout AA Bengtsson T Sirsjo A Fransen K. Role of NLRP3 and CARD8 in the regulation of TNF-alpha induced IL-1beta release in vascular smooth muscle cells. Int J Mol Med. (2012) 30:697–702. 10.3892/ijmm.2012.1026 22711073

[17] Weber A Wasiliew P Kracht M. Interleukin-1 (IL-1) pathway. Sci Signal. (2010) 3:cm1. 10.1126/scisignal.3105cm1 20086235

[18] Kim DH Bang E Arulkumar R Ha S Chung KW Park MH. Senoinflammation: a major mediator underlying age-related metabolic dysregulation. Exp Gerontol. (2020) 134:110891. 10.1016/j.exger.2020.110891 32114077

[19] Cagel M Grotz E Bernabeu E Moretton MA Chiappetta DA. Doxorubicin: nanotechnological overviews from bench to bedside. Drug Discov Today. (2017) 22:270–81. 10.1016/j.drudis.2016.11.005 27890669

[20] Bielak-Zmijewska A Wnuk M Przybylska D Grabowska W Lewinska A Alster O. A comparison of replicative senescence and doxorubicin-induced premature senescence of vascular smooth muscle cells isolated from human aorta. Biogerontology. (2014) 15:47–64. 10.1007/s10522-013-9477-9 24243065 PMC3905196

[21] Schuchardt M Tolle M Prufer J Prufer N Huang T Jankowski V. Uridine adenosine tetraphosphate activation of the purinergic receptor P2Y enhances in vitro vascular calcification. Kidney Int. (2012) 81:256–65. 10.1038/ki.2011.326 21956191

[22] Herrmann J Babic M Tolle M Eckardt KU Van Der Giet M Schuchardt M. A novel protocol for detection of senescence and calcification markers by fluorescence microscopy. Int J Mol Sci. (2020) 21:3475. 10.3390/ijms21103475 32423114 PMC7278918

[23] Lopes-Paciencia S Saint-Germain E Rowell MC Ruiz AF Kalegari P Ferbeyre G. The senescence-associated secretory phenotype and its regulation. Cytokine. (2019) 117:15–22. 10.1016/j.cyto.2019.01.013 30776684

[24] Niemi K Teirila L Lappalainen J Rajamaki K Baumann MH Oorni K. Serum amyloid a activates the NLRP3 inflammasome via P2X7 receptor and a cathepsin B-sensitive pathway. J Immunol. (2011) 186:6119–28. 10.4049/jimmunol.1002843 21508263

[25] De Zoete MR Palm NW Zhu S Flavell RA. Inflammasomes. Cold Spring Harb Perspect Biol. (2014) 6:a016287. 10.1101/cshperspect.a016287 25324215 PMC4292152

[26] Schuchardt M Prufer N Tu Y Herrmann J Hu XP Chebli S. Dysfunctional high-density lipoprotein activates toll-like receptors via serum amyloid a in vascular smooth muscle cells. Sci Rep. (2019) 9:3421. 10.1038/s41598-019-39846-3 30833653 PMC6399289

[27] Burtenshaw D Hakimjavadi R Redmond EM Cahill PA. Nox, reactive oxygen species and regulation of vascular cell fate. Antioxidants. (2017) 6:90. 10.3390/antiox6040090 29135921 PMC5745500

[28] Bernadotte A Mikhelson VM Spivak IM. Markers of cellular senescence. Telomere shortening as a marker of cellular senescence. Aging. (2016) 8:3–11. 10.18632/aging.100871 26805432 PMC4761709

[29] Duer M Cobb AM Shanahan CM. DNA damage response: a molecular lynchpin in the pathobiology of arteriosclerotic calcification. Arterioscler Thromb Vasc Biol. (2020) 40:e193–202. 10.1161/ATVBAHA.120.313792 32404005

[30] Mijit M Caracciolo V Melillo A Amicarelli F Giordano A. Role of p53 in the regulation of cellular senescence. Biomolecules. (2020) 10:420. 10.3390/biom10030420 32182711 PMC7175209

[31] Campisi J. Senescent cells, tumor suppression, and organismal aging: good citizens, bad neighbors. Cell. (2005) 120:513–22. 10.1016/j.cell.2005.02.003 15734683

[32] Orjalo AV Bhaumik D Gengler BK Scott GK Campisi J. Cell surface-bound IL-1alpha is an upstream regulator of the senescence-associated IL-6/IL-8 cytokine network. Proc Natl Acad Sci USA. (2009) 106:17031–36. 10.1073/pnas.0905299106 19805069 PMC2761322

[33] Acosta JC Banito A Wuestefeld T Georgilis A Janich P Morton JP. A complex secretory program orchestrated by the inflammasome controls paracrine senescence. Nat Cell Biol. (2013) 15:978–90. 10.1038/ncb2784 23770676 PMC3732483

[34] Burton DG Giles PJ Sheerin AN Smith SK Lawton JJ Ostler EL. Microarray analysis of senescent vascular smooth muscle cells: a link to atherosclerosis and vascular calcification. Exp Gerontol. (2009) 44:659–65. 10.1016/j.exger.2009.07.004 19631729

[35] Schuchardt M Herrmann J Henkel C Babic M Van Der Giet M Tolle M. Long-term treatment of azathioprine in rats induces vessel mineralization. Biomedicines. (2021) 9:327. 10.3390/biomedicines9030327 33806932 PMC8004774

[36] Chen GY Nunez G. Sterile inflammation: sensing and reacting to damage. Nat Rev Immunol. (2010) 10:826–37. 10.1038/nri2873 21088683 PMC3114424

[37] Roh JS Sohn DH. Damage-associated molecular patterns in inflammatory diseases. Immune Netw. (2018) 18:e27. 10.4110/in.2018.18.e27 30181915 PMC6117512

[38] Wen C Yang X Yan Z Zhao M Yue X Cheng X. Nalp3 inflammasome is activated and required for vascular smooth muscle cell calcification. Int J Cardiol. (2013) 168:2242–7. 10.1016/j.ijcard.2013.01.211 23453445

[39] Evavold CL Ruan J Tan Y Xia S Wu H Kagan JC. The pore-forming protein gasdermin d regulates interleukin-1 secretion from living macrophages. Immunity. (2018) 48:35–44 e36. 10.1016/j.immuni.2017.11.013 29195811 PMC5773350

[40] Rea IM Gibson DS Mcgilligan V Mcnerlan SE Alexander HD Ross OA. Age and age-related diseases: role of inflammation triggers and cytokines. Front Immunol. (2018) 9:586. 10.3389/fimmu.2018.00586 29686666 PMC5900450

[41] Xu D Zeng F Han L Wang J Yin Z Lv L. The synergistic action of phosphate and interleukin-6 enhances senescence-associated calcification in vascular smooth muscle cells depending on p53. Mech Ageing Dev. (2019) 182:111124. 10.1016/j.mad.2019.111124 31376399

[42] Han L Zhang Y Zhang M Guo L Wang J Zeng F. Interleukin-1beta-induced senescence promotes osteoblastic transition of vascular smooth muscle cells. Kidney Blood Press Res. (2020) 45:314–30. 10.1159/000504298 32126555

[43] Tsai IC Pan ZC Cheng HP Liu CH Lin BT Jiang MJ. Reactive oxygen species derived from NADPH oxidase 1 and mitochondria mediate angiotensin II-induced smooth muscle cell senescence. J Mol Cell Cardiol. (2016) 98:18–27. 10.1016/j.yjmcc.2016.07.001 27381955

[44] Sun M Chang Q Xin M Wang Q Li H Qian J. Endogenous bone morphogenetic protein 2 plays a role in vascular smooth muscle cell calcification induced by interleukin 6 in vitro. Int J Immunopathol Pharmacol. (2017) 30:227–37. 10.1177/0394632016689571 28134597 PMC5815263

[45] Kurozumi A Nakano K Yamagata K Okada Y Nakayamada S Tanaka Y. IL-6 and sIL-6R induces STAT3-dependent differentiation of human VSMCs into osteoblast-like cells through JMJD2B-mediated histone demethylation of RUNX2. Bone. (2019) 124:53–61. 10.1016/j.bone.2019.04.006 30981888

[46] Regent A Ly KH Lofek S Clary G Tamby M Tamas N. Proteomic analysis of vascular smooth muscle cells in physiological condition and in pulmonary arterial hypertension: toward contractile versus synthetic phenotypes. Proteomics. (2016) 16:2637–49. 10.1002/pmic.201500006 27458111

[47] Ridker PM Everett BM Thuren T Macfadyen JG Chang WH Ballantyne C. Antiinflammatory therapy with canakinumab for atherosclerotic disease. N Engl J Med. (2017) 377:1119–31. 10.1056/NEJMoa1707914 28845751

