# Supplementary material for: Stressor-Induced “Inflammaging” of Vascular Smooth Muscle Cells via Nlrp3-Mediated Pro-inflammatory Auto-Loop
Source: Front Cardiovasc Med. 2021 Dec 20;8:752305. doi: 10.3389/fcvm.2021.752305 (PMC8720922; doi:10.3389/fcvm.2021.752305)
Supplement: Supplementary file 1 [file Data_Sheet_1.docx]

***Supplementary Material***

1. **Quantitative real-time PCR**

The oligonucleotide sequences are summarized in Supplementary Table 1.

**Supplementary Table 1.** **Oligonucleotide sequences (rat)**

| **Gene** | **Fwd 5´-3´**  **Rev 5´-3´** | **Company** | **Reference** |
| --- | --- | --- | --- |
| Acta2 | ACC ATC GGG AAT GAA CGC TT  CTG TCA GCA ATG CCT GGG TA | Tib Mol | (1) |
| β-Actin | TCG CTG ACA GGA TGC AGA AG  CTC AGG AGG AGC AAT GAT CTT GAT | Tib Mol | Primer Blast |
| Bmp-2 | ACT TCC CGA CGC TTC TTC TTC A  GGC CAC TTC CAC CAC AAA CC | Tib Mol | (2) |
| Cbfa1 | GCC GGG AAT GAT GAG AAC TA  GGA CCG TCC ACT GTC ACT TT | Tib Mol | Primer Blast |
| Cnn1 | GCC CAG AAA TAC GAC CAC CA  CCG GCTGGA GCT TGT TGA TA | Tib Mol | (1) |
| Gapdh | TGC CAA GTA TGA CAT CAA GAA G  AGC CCA GGA TGC CCT TTA GT | Tib Mol | Primer Blast |
| Il-1β | GCT ATG GCA ACT GTC CCT GA  AAG GGC TTG GAA GCA ATC CTT A | Tib Mol | Primer Blast |
| Il-6 | CTG GTC TTC TGG AGT TCC GT  TGG TCC TTA GCC ACT CCT TCT | Tib Mol | Primer Blast |
| p16 | GGC TTC ACC AAA CGC CCC GA  GCT GCT TTG GGG GTT GGC CT | Tib Mol | Primer Blast |
| p21 | TAT GTA CCA GCC ACA GGC AC  ATC GGC GCT TGG AGT GAT AG | Tib Mol | Primer Blast |
| Myh11 | CAC TGA GAG CAA TGA GGC CA  TCT GAG TCC CGA GCA TCC AT | Tib Mol | (1) |
| Nlrp3 | CCA GGG CTC TGT TCA TTG  CCT TGG CTT TCA CTT CG | Tib Mol | (3) |
| Asc | TTA TGG AAG AGT CTG GAG CTG TG  GCA ATG AGT GCT TGC CTG TG | Tib Mol | Primer Blast |
| Caspase-1 | GGA GCT TCA GTC AGG TCC ATC  CTT GAG GGA ACC ACT CGG TC | Tib Mol | Primer Blast |
| Tlr2 | GGT CTC CAG GTC AAA TCT CAG AGG A  CGG AGG TTC ACA CAG GCT CGC | Tib Mol | Primer Blast |
| Tlr4 | GGC ATC ATC TTC ATT GTC CTT G  AGC ATT GTC CTC CCA CTC G | Tib Mol | (4) |
| Nox1 | CTG CTC TCC TTC CTG AGG GGC ACC TGC T  GAC AAT CCC CCC CAG GCC ATG GAT CCC TA | Tib Mol | (5) |

1. **Cytotoxicity**

Cytotoxicity was assessed with the CytoTox-Glo™ Cytotoxicity Assay (Promega) according to the manufacturer’s instruction. VMSC were seeded in 96-well plates, serum starved for 24 h and stimulated for 48 h or 72 h. Luminescence was measured with the Mithras LB940 device and the MicroWin software (version 5.22, Berthold Technologies).

We found no statistically significant induction of cytotoxicity after stimulation with DOX, Il-6 and Il-1β. Nevertheless, although the effect does not reach statistical significance, the results indicate a dose-dependent cytotoxic effect of DOX after stimulation for 72 h that is also visible via light microscopy.


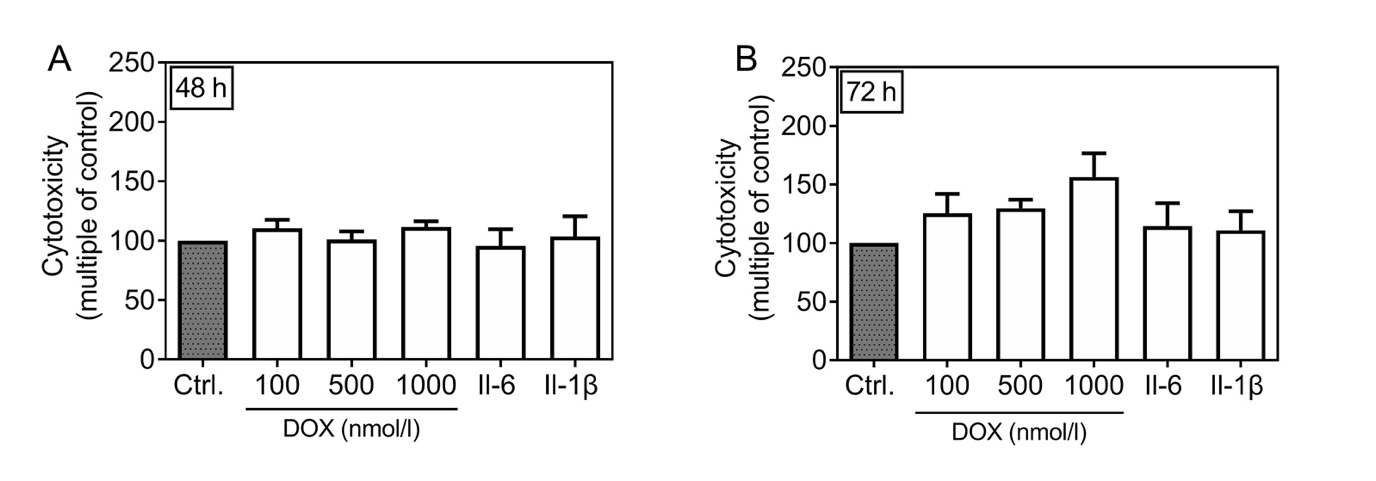


**Supplementary Figure 1. Cytotoxicity upon stimulation with DOX, Il-6 and Il-1β**

VSMCs were stimulated as indicated with DOX, 100 ng/ml Il-6 and 100 ng/ml Il-1β for 48 h (A) or 72 h (B). Cytotoxicity was determined using the CytoTox-Glo™ Cytotoxicity Assay. Data represent mean±SEM, n≥3.

1. **Western Blots after stimulation with DOX and Il-1β**

Suppl. Figure 2 shows the full representative multi-plex Western Blot for Figure 5D. Supplementary Figure 3 shows the full representative Western Blot for Cbfa1 from Figure 5A. Supplementary Figure 4 shows the full representative Western Blot for Actin from Figure 5B. The unspecific bands most likely comes from the secondary antibody.


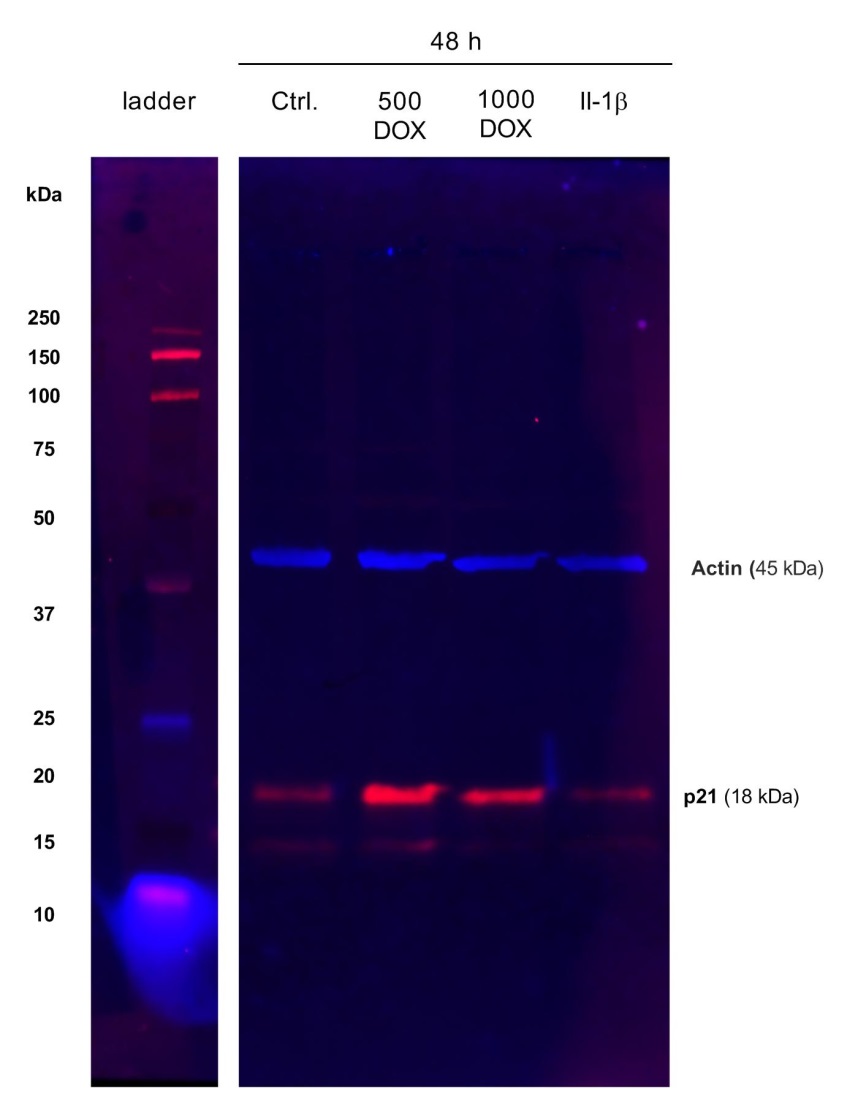


**Supplementary Figure 2. p21 WB after stimulation with DOX and Il-1β**

VSMCs were stimulated as indicated with 500 nmol/l or 1000 nmol/l DOX or 100 ng/ml Il-1β for 48 h as indicated. Protein content of p21 and Actin were identified with Western Blot. The Western Blot is a full representative image of n=3.


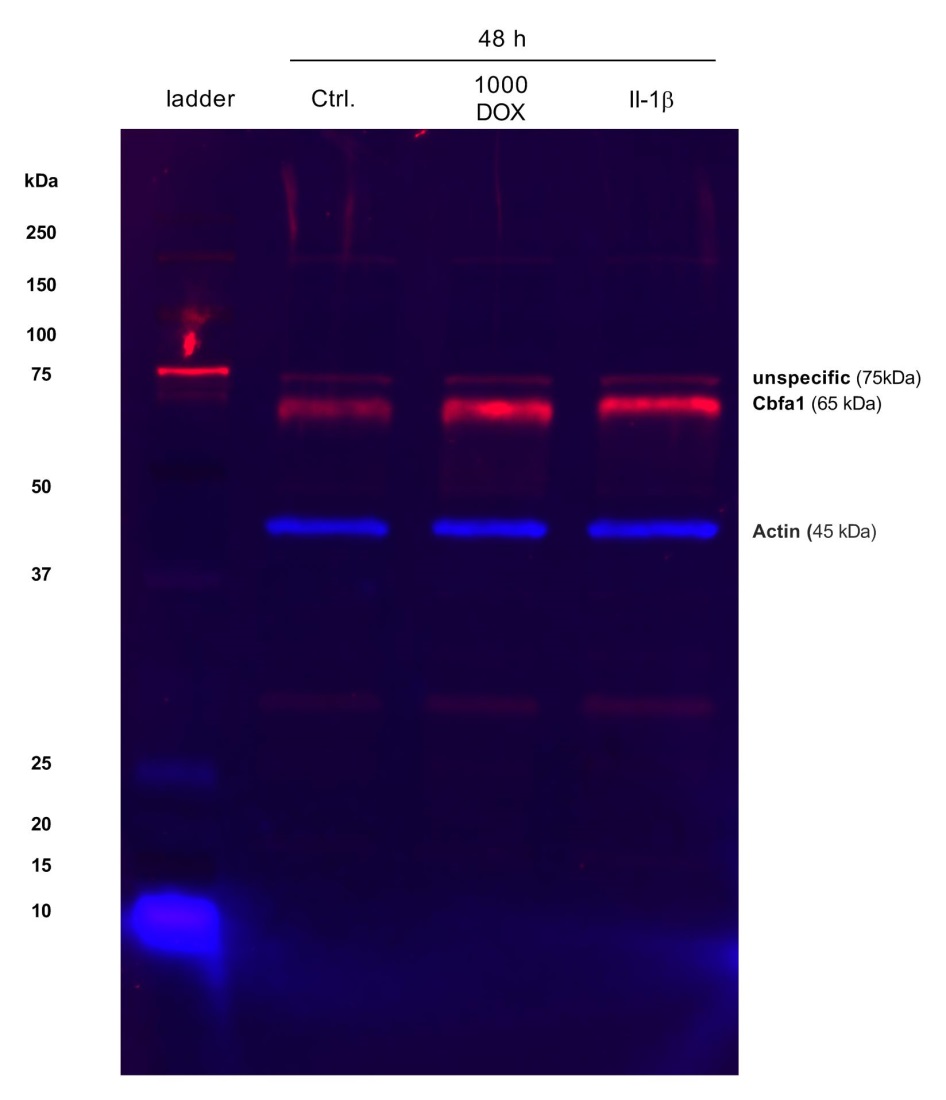


**Supplementary Figure 3. Cbfa1 and Actin WB after stimulation with DOX and Il-1β**

VSMCs were stimulated as indicated with 1000 nmol/l DOX or 100 ng/ml Il-1β for 48 h. Protein content of Cbfa1 and Actin were identified with Western Blot. The Western Blot is a full representative image of n=3.


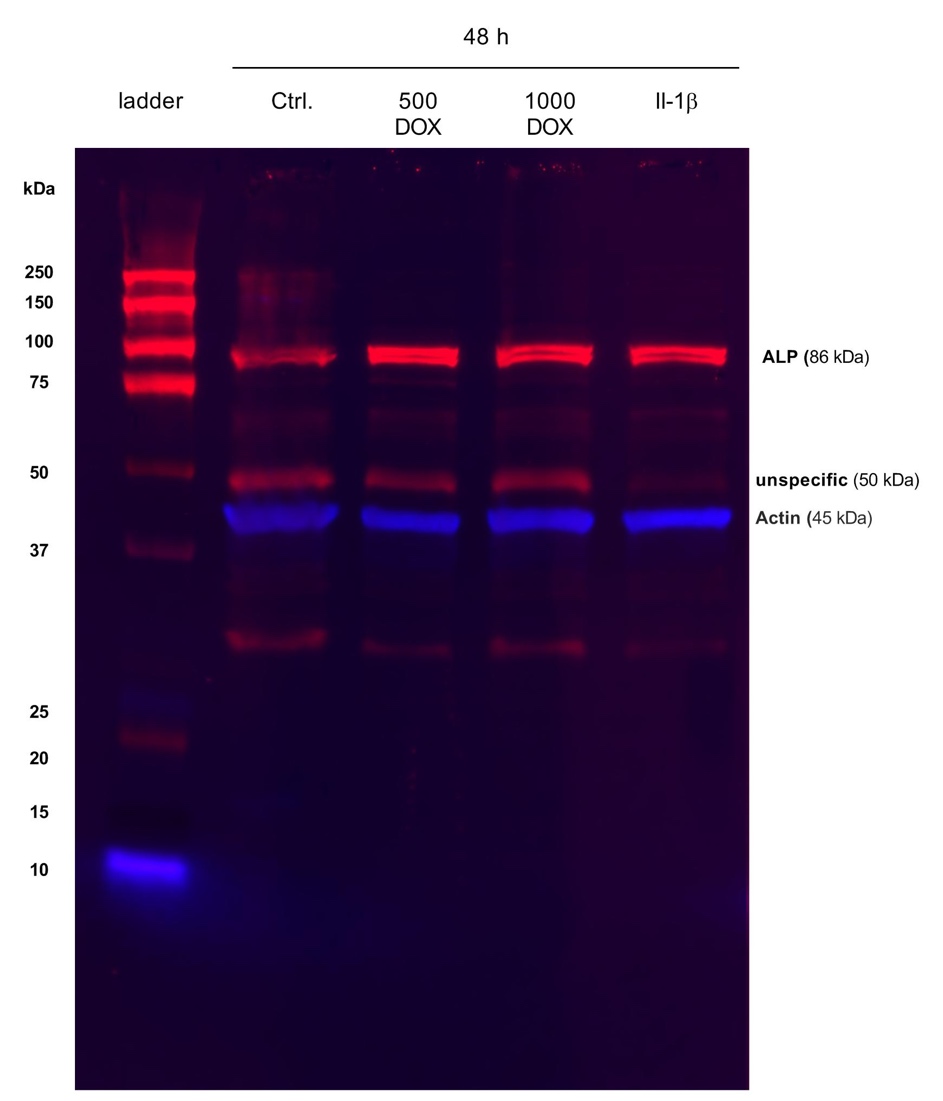


**Supplementary Figure 4. Alp and Actin WB after stimulation with DOX and Il-1β**

VSMCs were stimulated as indicated with 1000 nmol/l DOX or 100 ng/ml Il-1β for 48 h. Protein content of Alp and Actin were identified with Western Blot. The Western Blot is a full representative image of n=3.

1. **Gene Expression after Il-6 Stimulation**

Gene expression was measured after stimulation with Il-6 after 48h in quiescence medium.


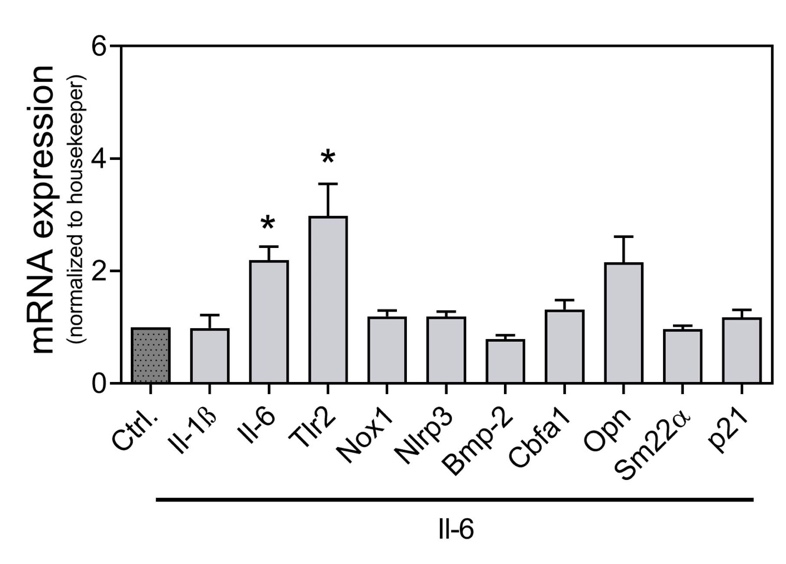


**Supplementary Figure 5. Gene Expression upon stimulation with Il-6**

VSMCs were stimulated as indicated with 100 ng/ml Il-6 for 48 h and mRNA expression was measured with quantitative real-time PCR. Measured thresholds of the respective targets were normalized to the corresponding average threshold of housekeepers Actin and Gapdh before normalization to control. Data represent mean±SEM, n≥3.

1. **RNA *in situ* hybridization**

VSMCs were seeded in LabTec chamber slides (Thermo Fisher). Cells were serum starved for 24 h and stimulated for 48 h. Staining of mRNA was performed with the RNAscope® Fluorescent Multiplex Assay (ACD) according to the manufacturer’s protocol. The materials used are given in suppl. Table 2.

**Supplementary Table 2.** **Components for RNA *in situ* hybridization**

| **Material** | **Company** | **Order Number** |
| --- | --- | --- |
| RNAscope™ Target Retrieval Reagents | ACD Bio | 322000 |
| RNAscope™ Fluorescent Multiplex Reagent Kit | ACD Bio | 320850 |
| RNAscope™ Wash Buffer Reagents | ACD Bio | 310091 |
| ImmEdge™ Hydrophobic Barrier Pen | ACD Bio | 310018 |
| RNAscope™ Probe - Rn-Cdkn1a | ACD Bio | 423851-C3 |
| RNAscope™ Probe - Rn-Spp1 | ACD Bio | 405441 |
| RNAscope™ Probe - Rn-Bmp2 | ACD Bio | 581071 |
| Prolong Diamond antifade medium | Thermo Fisher Scientific | P10144 |

Cells were imaged with an Axiovert 200M microscope. The channel Atto647 was quantified with Zen2 software (Blue edition, Zeiss) using a 3-Sigma-threshold approach. Nuclei area was calculated with Zen2 software (Zeiss) with manual threshold determination. Due to background and channel cross talk, the channels Alexa488 and Atto550 were analyzed manually with ImageJ. Briefly, pictures were exported from Zen in jpg-format, inverted and targets were identified, marked manually, and counted.

1. **γH2A.X staining**

VSMCs were seeded in LabTec chamber slides (Thermo Fisher). Cells were serum starved for 24 h and stimulated for 48 h. After permeabilization with Triton X (0,1%) cells were stained with (sc-101696) 1:500 in 10% RotiBlock/PBS for 1 h at RT, followed by incubation with the Alexa Fluor 555-coupled secondary antibody (Invitrogen, A-21429) 1:1,000 in 10% RotiBlock/PBS for 1 h at RT. Nuclei were counterstained with Hoechst33342 (Thermo Fisher) according to the manufacturer’s protocol. Cells were imaged with an Axiovert 200M microscope. The channel “Alexa 555” was quantified with Zen2 software (Blue edition, Zeiss) using a 3 Sigma threshold approach. The cell core area was determined with Zen2 software with manual threshold determination.

1. **SA-**β**-Gal Staining**

VSMCs were seeded in µ-Slides slides (Ibidi). Cells were serum starved for 24 h and stimulated for 72 h. After stimulation, cells were washed with PBS, fixed for 3 min at room temperature with 4% Formalin and washed with PBS. A 2 µM working solution of SPiDER- β - Gal in 1:5 with ultrapure water diluted McIlvaine buffer was incubated for 30min at 37°C. Afterwards cells were washed, counterstained with Hoechst 33342 (Thermo Fisher) according to the manufacturer’s protocol, covered with PBS and immediately imaged. The channel “BODIPY R6G” was quantified with Zen2 software (Blue edition, Zeiss) using a 3 Sigma threshold approach. The cell core area was determined with Zen2 software with manual threshold determination.

**Supplementary Table 3. Representative Images of RNA *in situ* hybridization,** γ**H2A.X staining and** β**-Gal staining upon DOX stimulation**

VSMCs were stimulated with 1000 nmol/l DOX, for 48 h (RNA Scope and γH2A.X) staining or 72 h (SA-β-Gal). VSMCs were stained with RNA *in situ* hybridization technique for mRNA expression of Bmp-2, Opn and p21, for formation of γH2A.X immunohistochemically or for the formation of SA-β-Gal with Spider. Representative images are shown for n≥3 experiments. The scale bar indicates a 20 µm section.

|  | **Ctrl.** | **DOX** |
| --- | --- | --- |
| **Bmp-2** | 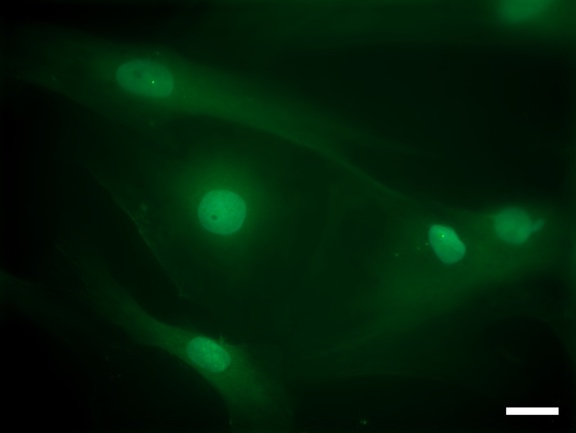 | 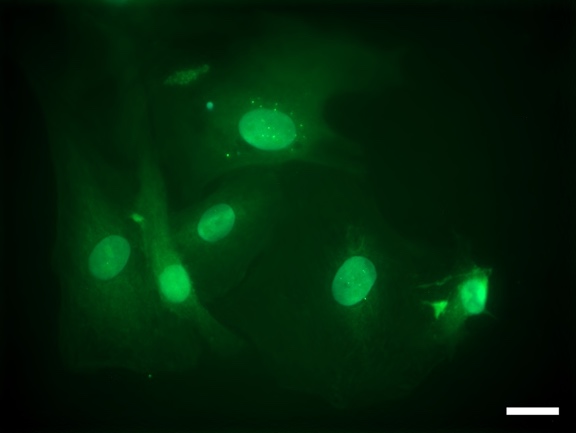 |
| **Opn** | 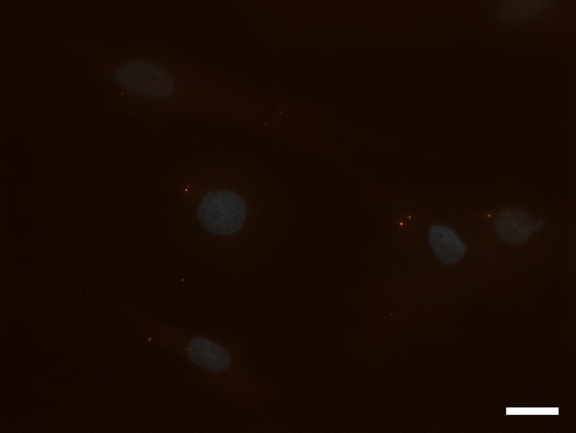 | 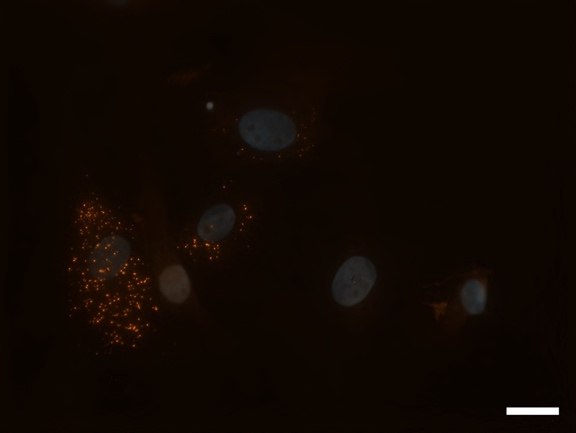 |
| **p21** | 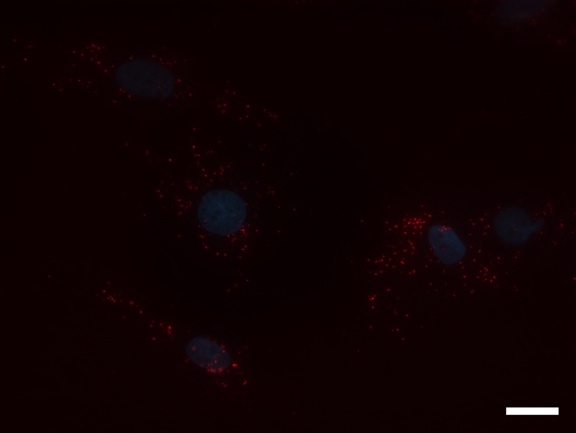 | 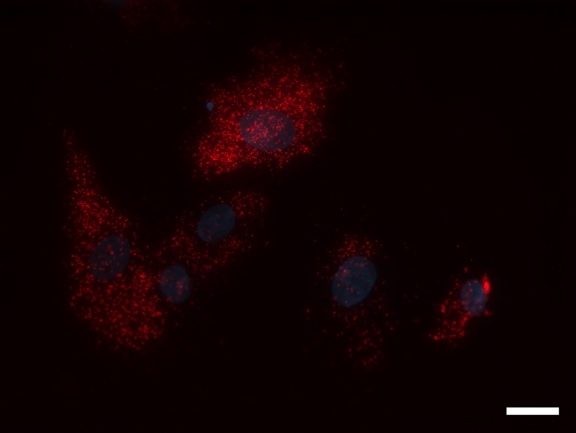 |
| γ**H2A.X** | 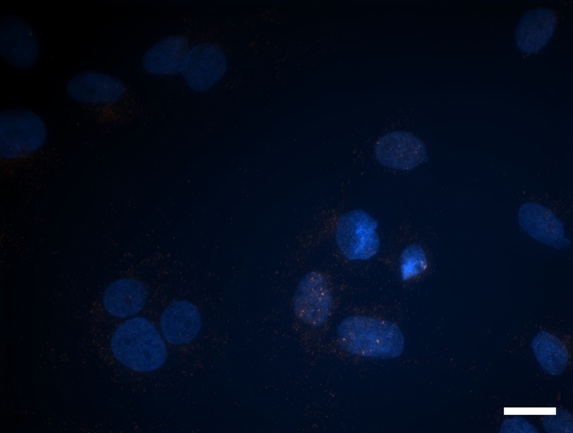 | 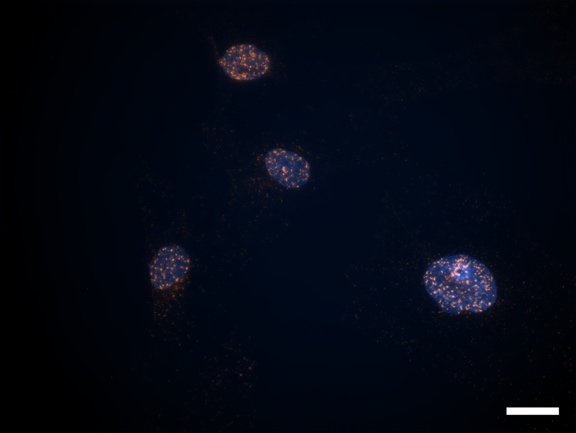 |
| **SA-β-Gal** | 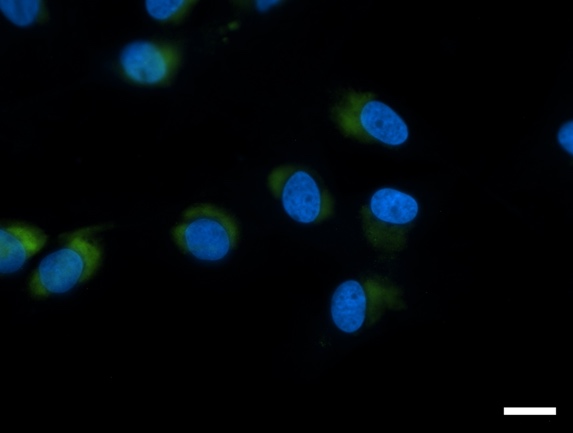 | 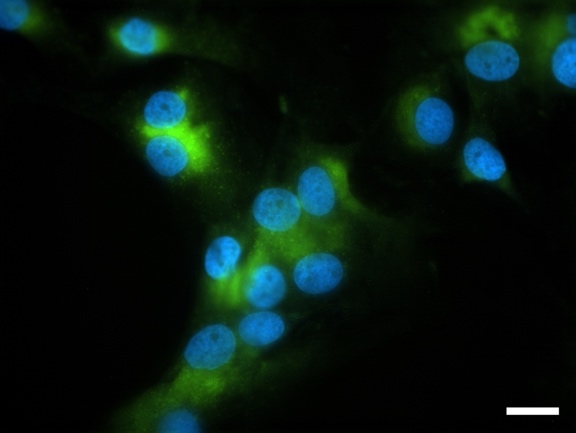 |

**Supplementary Table 4. Representative Images of RNA *in situ* hybridization,** γ**H2A.X staining and SA-**β**-Gal staining upon Il-6 and Il-1**β **stimulation**

VSMCs were stimulated with 100 ng/ml Il-1β or 100 ng/ml Il-6 for 48 h (RNA Scope and γH2A.X staining) or 72 h (SA-β-Gal). VSMCs were stained with RNA *in situ* hybridization technique for mRNA expression of Bmp-2, Opn and p21, for formation of γH2A.X immunohistochemically or for the formation of SA-β-Gal with Spider. Representative images are shown for n≥3 experiments. The scale bar indicates a 20 µm section.

|  | **Ctrl.** | **Il-1β** | **Il-6** |
| --- | --- | --- | --- |
| **Bmp-2** | 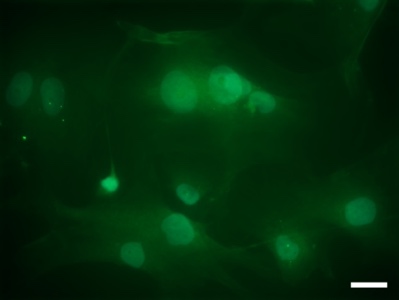 | 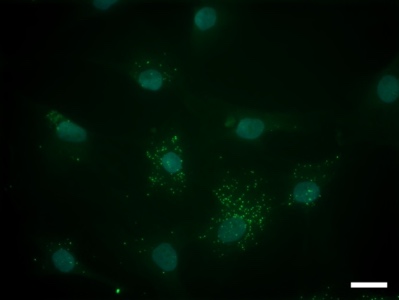 | 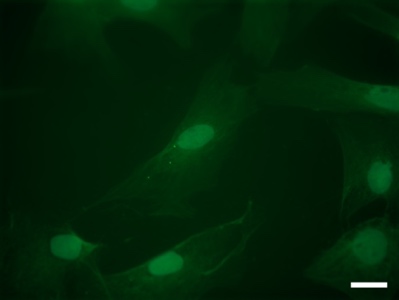 |
| **Opn** | 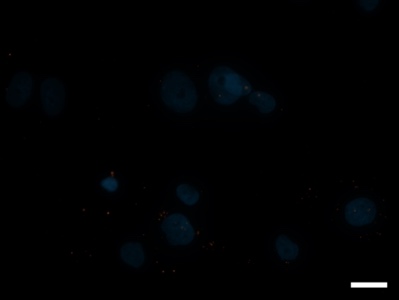 | 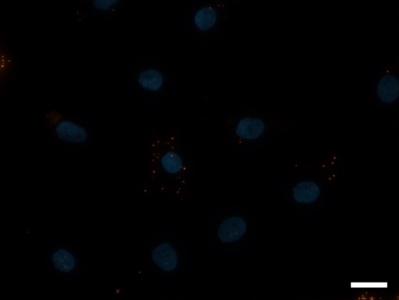 | 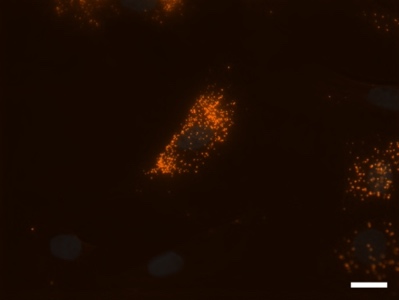 |
| **p21** | 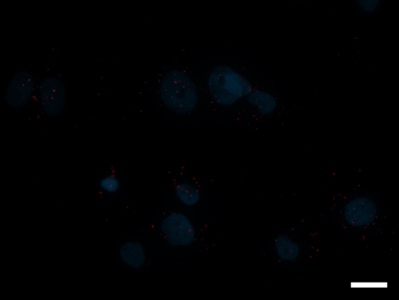 | 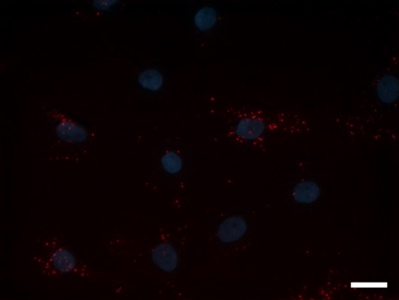 | 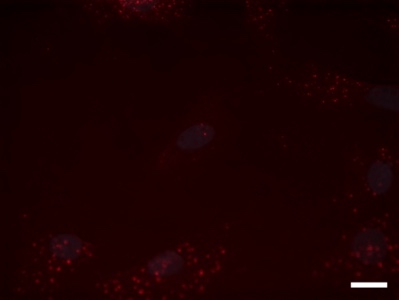 |
| γ**H2A.X** | 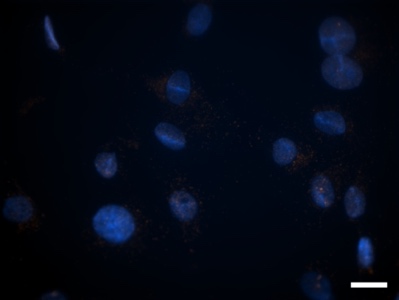 | 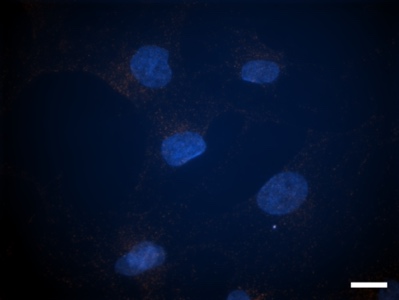 | 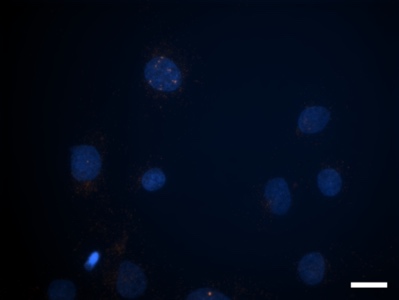 |
| **SA-β-Gal** | 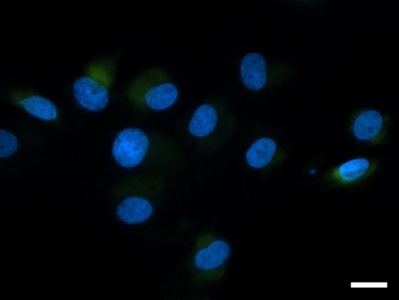 | 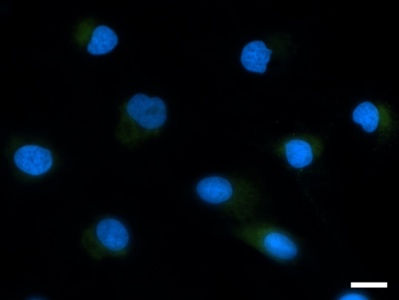 | 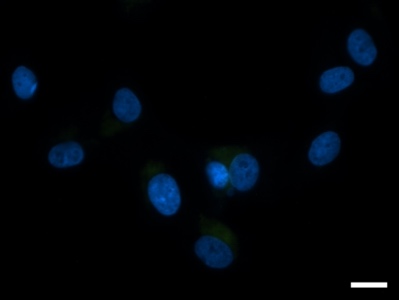 |

1. ***DHE staining***

The detailed staining procedure is described in the main text. Cells were imaged with an Axiovert 200M microscope. The channel “ethidium homodimer” was quantified with Zen2 software (Blue edition, Zeiss) using an Otsu threshold approach. The detected intensity of the channel was normalized to area identified by the threshold procedure.

**Supplementary Table 5. Representative Images of DHE staining**

VSMCs were stimulated with 1000 nM DOX, 100 ng/ml Il-1β for 30 min. VSMCs were stained with DHE. Pictures show a representative experiment of n≥3.

|  |  |  |  |
| --- | --- | --- | --- |
| **Ctrl.** | 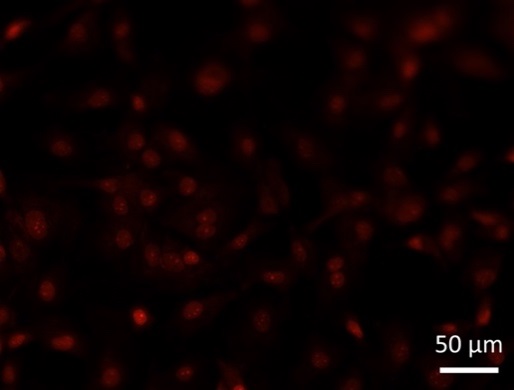 | **Ctrl.** | 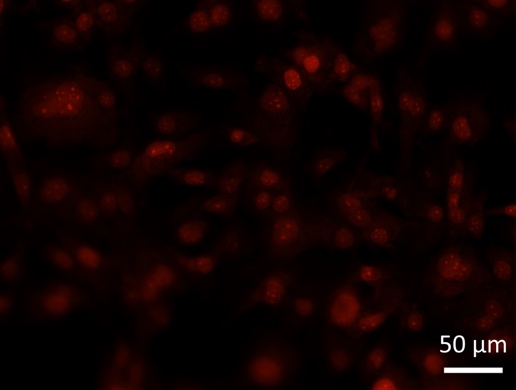 |
| **DOX** | 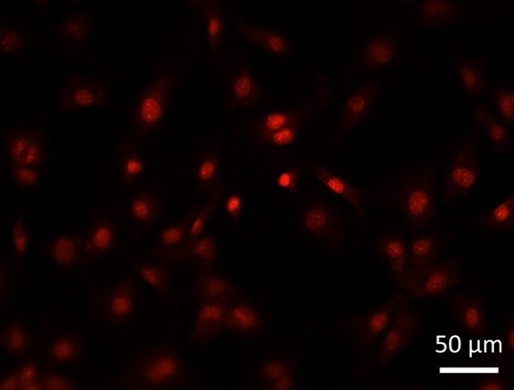 | **Il-1β** | 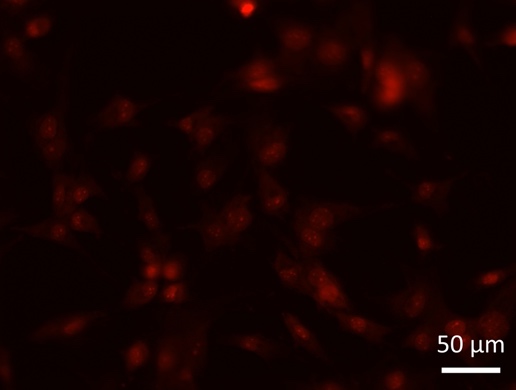 |

**References**

(1) Jung, Y., Lee, H.S., Ha, J.M., Jin, S.Y., Kum, H.J., Vafaeinik, F., Ha, H.K., Song, S.H., Kim, C.D., and Bae, S.S. (2021). Modulation of Vascular Smooth Muscle Cell Phenotype by High Mobility Group AT-Hook 1. *J Lipid Atheroscler* 10**,** 99-110.

(2) Maciel, T.T., Melo, R.S., and Campos, A.H. (2009). The bone morphogenetic protein antagonist gremlin promotes vascular smooth muscle cell apoptosis. *J Vasc Res* 46**,** 325-332.

(3) Liu, H.D., Li, W., Chen, Z.R., Hu, Y.C., Zhang, D.D., Shen, W., Zhou, M.L., Zhu, L., and Hang, C.H. (2013). Expression of the NLRP3 inflammasome in cerebral cortex after traumatic brain injury in a rat model. *Neurochem Res* 38**,** 2072-2083.

(4) Liu, N., Liu, J., Ji, Y., Lu, P., Wang, C., and Guo, F. (2010). C-Reactive Protein Induces TNF-alpha Secretion by p38 MAPK-TLR4 Signal Pathway in Rat Vascular Smooth Muscle Cells. *Inflammation*.

(5) San Martin, A., Foncea, R., Laurindo, F.R., Ebensperger, R., Griendling, K.K., and Leighton, F. (2007). Nox1-based NADPH oxidase-derived superoxide is required for VSMC activation by advanced glycation end-products. *Free Radic Biol Med* 42**,** 1671-1679.
